# Supplementary material for: Loss-of-function variants in the CAPN1 activator CD99L2 cause X-linked spastic ataxia
Source: Nat Commun. 2026 Feb 14;17:1698. doi: 10.1038/s41467-026-69337-9 (PMC12910011; doi:10.1038/s41467-026-69337-9)
Supplement: Supplementary file 1 — Supplementary Information [file 41467_2026_69337_MOESM1_ESM.pdf]

Supplementary information

Benita Menden, Rana D. Incebacak Eltemur, et al. **Loss-of-function variants in the CAPN1 activator *CD99L2* cause X-linked spastic ataxia**.

In the format provided by the authors and unedited

Table of contents

1. Supplementary tables ... 4

Supplementary Table 1: HPO-based movement disorder subgroups. ... 4

Supplementary Table 2: Primary antibodies for western blotting. ... 5

2. Supplementary figures and figure legends ... 6

Supplementary Figure 1: Diagnostic testing strategy, clinical phenotypes as well as spectrum of affected genes found in the cohort. ... 6

Supplementary Figure 2: Detailed description of ES cohort. ... 7

Supplementary Figure 3: Subgroups, age-dependent outcome and findings in ES cohort. ... 8

Supplementary Figure 4: Detailed description of GS cohort. ... 10

Supplementary Figure 5: Identification of factors predictive of diagnostic yield. ... 11

Supplementary Figure 6: Distribution of symptoms in CD99L2 patients. ... 12

Supplementary Figure 7: Additional pedigrees of investigated families and detections of CD99L2 protein in patient-derived primary fibroblasts. ... 13

Supplementary Figure 8: Analysis of ubiquitination as a major post-translational modification of CD99L2. ... 14

Supplementary Figure 9: CD99L2 binds and activates CAPN1. ... 16

Supplementary Figure 10: CAST overexpression and pharmacological inhibitor treatment counteracts CD99L2-mediated calpain activation, while increasing CD99L2 levels. ... 17

Supplementary Figure 11: CD99L2 is a target of calpain-mediated cleavage. ... 18

Supplementary Figure 12: Analysis of CD99L2 truncation constructs. ... 19

Supplementary Figure 13: Transcriptomic analysis of CD99L2 patient fibroblasts. ... 20

3. Illustrative examples for the added diagnostic yield of genome sequencing ... 22

Example 1: Improved variant calling due to longer read length in genome sequencing ... 22

Example 2: Second allele in noncoding first exon missed by exome sequencing in a recessive disease ... 24

Example 3: Second allele with copy-neutral inversion in recessive disease ... 26

Example 4: Homozygous copy-neutral inversion in recessive disease missed by exome analysis 27

Example 5: Detection of a repeat expansion in FXN via GS in an atypical case of Friedreich ataxia ... 28

4. Case reports *CD99L2* families ... 29

Family 1, individual II.1 [c.51del, p.Thr18ProfsTer19] ... 29

Family 2, individual III.1 [deletion exons 9-11] ... 29

Family 3, individual III.1 [c.281_282del, p.Arg94MetfsTer24] ... 29

Family 4, individual II.4 [c.382C>T, p.Arg128Ter] ... 29

Family 5, individual III.1 [c.382C>T, p.Arg128Ter] ... 30

Family 6, individual III.1 and III.3 [c.382C>T, p.Arg128Ter] 30
Family 7, individual II.4 [c.382C>T, p.Arg128Ter] 30
Family 8, individual III.1 [c.382C>T, p.Arg128Ter] 30
Family 9, individual II.2 [c.382C>T, p.Arg128Ter] 31
Family 10, individual III.2 [c.382C>T, p.Arg128Ter] 31
Family 11, individual III.2 [c.391C>T, p.Arg131Ter] 31
Family 12, individual III.3 [c.391C>T, p.Arg131Ter] 31
Family 13, individuals III.1-4 and II.2-3 [c.467del, p.Gly156ValfsTer71] 31
Family 14, individual III.2 [deletion exons 8, 9] 32
Family 15, individual II.2 [deletion exons 8, 9] 32
Family 16, individual II.2 [c.535G>C, p.Gly179Arg] 32
Family 18, individual I.2 [c.655+1G>A, p.?] 33
Family 18, individual II.2 [c.655+1G>A, p.?] 33
Family 18, individual II.3 [c.655+1G>A, p.?] 34
Family 19, individual III.5 [c.655+1G>A, p.?] 34
Family 20, individual II.2 und II.3 [c.655+3A>C, p.?] 35
5. Consortia and associated partners 36
Solve-RD consortium 36
Associated partners 37

1. Supplementary tables

Supplementary Table 1: HPO-based movement disorder subgroups.

**Ataxia**	**Spasticity**	**Dystonia**
ataxia	spasticity	torticollis
gait ataxia	lower limb spasticity	torsion dystonia
truncal ataxia	spastic gait	oromandibular dystonia
vestibular areflexia	progressive spasticity	writer's cramp
spastic ataxia	spastic paraplegia	blepharospasm
sensory ataxia	spastic paraparetic gait	limb dystonia
progressive truncal ataxia	spastic paraparesis	craniofacial dystonia
progressive gait ataxia	progressive spastic quadriplegia	generalized dystonia
progressive cerebellar ataxia	upper limb spasticity	lingual dystonia
optic ataxia	spasticity of pharyngeal muscles	dystonia
nonprogressive cerebellar ataxia	spasticity of facial muscles	laryngeal dystonia
limb ataxia	spastic/hyperactive bladder	paroxysmal dystonia
episodic ataxia	spastic tetraparesis	axial dystonia
cerebellar ataxia associated with quadrupedal gait	spastic tetraplegia	focal dystonia
	spastic hemiparetic gait	orofacial action-specific dystonia induced by speech
	spastic hemiparesis	dystonic gait
	spastic dysarthria	arm dystonia
	spastic diplegia	hemidystonia
	spastic ataxia	leg dystonia
	slowly progressive spastic quadriparesis	
	progressive spastic paraplegia	
	progressive spastic paraparesis	

Supplementary Table 2: Primary antibodies for western blotting.

Antibody/target	Host	Dilution	Clone/catalog no.	Vendor
β-actin	mouse	1:5000	AC-15/A5441	Sigma-Aldrich
Ataxin-3	mouse	1:5000	1H9/MAB5360	Merck
Calnexin	rabbit	1:1000	C4731	Sigma-Aldrich
CAPN1	rabbit	1:1000	ab39170	Abcam
CAPN2	mouse	1:1000	1E1F10/66977-1-Ig	Proteintech
CAPN2	rabbit	1:500	ab39165	Abcam
CAPN10	rabbit	1:1000	ab28220	Abcam
CSS1	mouse	1:500	P1/MAB3083	Merck
CAST	rabbit	1:1000*	#4146S	Cell Signaling
CD99L2 (C-terminal)	rabbit	1:500	HPA061400	Sigma-Aldrich
CD99L2 (N-terminal)	rabbit	1:500	HPA038783	Sigma-Aldrich
FLAG	rabbit	1:1000	F7425	Sigma-Aldrich
GAPDH	mouse	1:2000	0411/sc-47724	Santa Cruz
GAPDH	rabbit	1:4000	10494-1-AP	Proteintech
LC3B	rabbit	1:1000*	#2775S	Cell Signaling
c-myc	mouse	1:200	9E10/sc-40	Santa Cruz
myc-tag	rabbit	1:1000*	71D10/#2278S	Cell Signaling
myc-tag	mouse	1:1000*	9B11/#2276S	Cell Signaling
O-GlcNAc	mouse	1:1000	RL2/sc-59624	Santa Cruz
OGT	mouse	1:1000	sc-74546	Santa Cruz
OGA	mouse	1:1000	sc-376429	Santa Cruz
α-spectrin	mouse	1:1000	AA6/MAB1622	Merck
ubiquitin (K48-linkage-specific)	rabbit	1:1000*	D9D5/#8081	Cell Signaling

All antibodies were diluted in 1× TBS with 0.1% (v/v) Tween-20 and 0.02% NaN_3. *Antibody dilutions were supplemented with 5% (w/v) bovine serum albumin.

2. Supplementary figures and figure legends

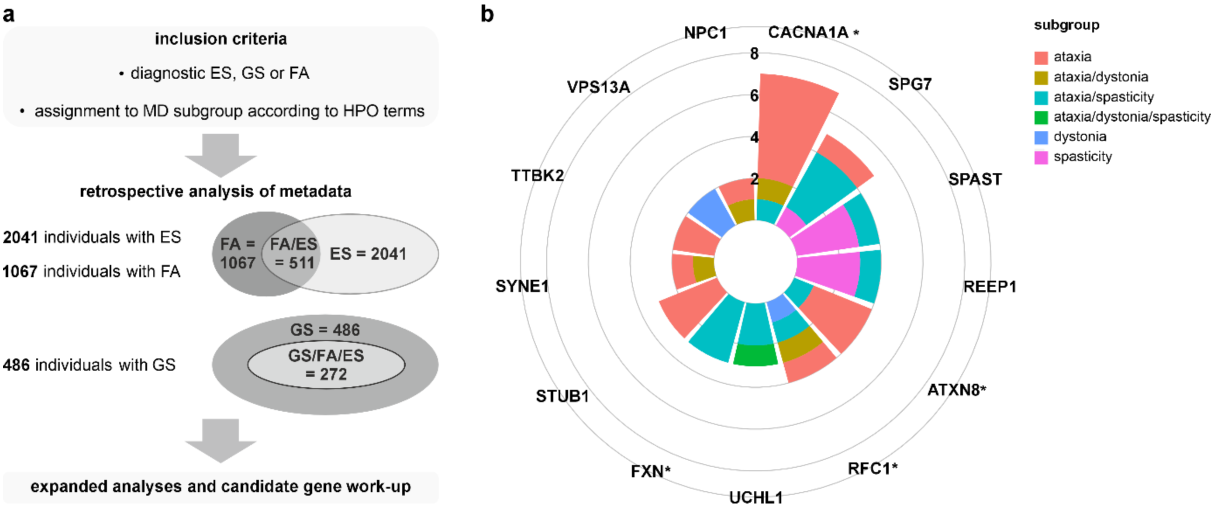

Supplementary Figure 1: Diagnostic testing strategy, clinical phenotypes as well as spectrum of affected genes found in the cohort.

a, Evolution of the diagnostic approach over a 6-year period with number of fragment analyses (FA), exome sequencing (ES), and genome sequencing (GS) performed. Early in the observation period, FA was performed first, followed by panel analysis (not recorded) or ES. A genome-first strategy was increasingly implemented in routine care in the further course, with indication for testing and discussion of results in multidisciplinary teams. Consented datasets were shared with the pan-European rare disease initiative Solve-RD. **b,** Representation of genes affected more than once in solved cases in the GS cohort and assignment of cases to different clinical subgroups. Genes carrying repeat expansions are marked with *. Source data are provided as a Source Data file.

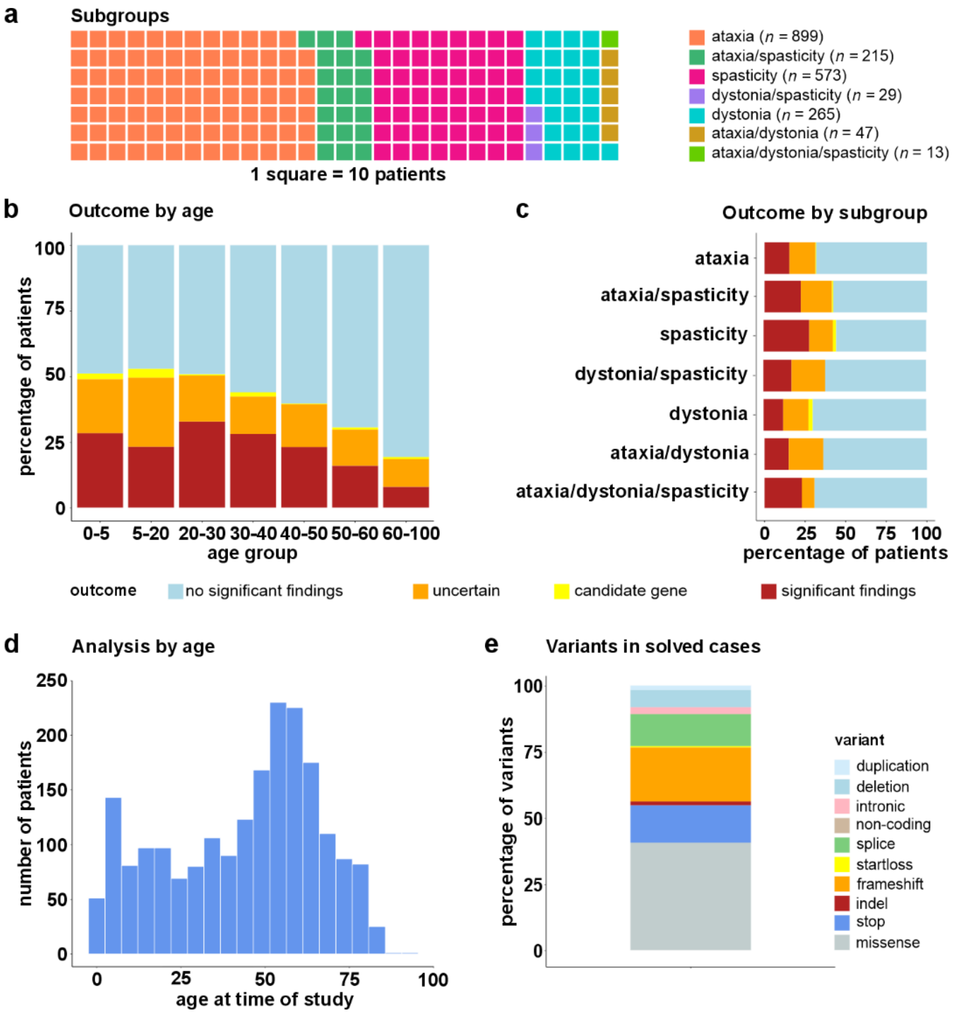

Supplementary Figure 2: Detailed description of ES cohort.

a, Distribution of HPO-based assignment to movement disorder subgroups. **b,** Percentual outcome by age for the age groups 0-5 y (*n* = 141, significant findings 28.4%), 5-20 y (*n* = 293, significant findings 23.2%), 20-30 y (*n* = 171, significant findings 32.8%), 30-40 y (*n* = 189, significant findings 28%), 40-50 y (*n* = 277, significant findings 23.1%), 50-60 y (*n* = 451, significant findings 16%), 60-100 y (*n* = 519, significant findings 7.9%). Age refers to age at diagnosis. **c,** Percentual outcome for the subgroups ataxia (15.4%), ataxia/spasticity (22.3%), spasticity (28.1%), dystonia/spasticity (17.2%), dystonia (12.1%), ataxia/dystonia (14.9%), ataxia/dystonia/spasticity (23.1%). **d,** Age distribution (median age: 49 y, IQR: 36 y). Age refers to age at diagnosis. **e,** Variants (ACMG class 4/5) in solved cases: duplications (1.6%), deletions (6.6%), intronic variants (2.5%), non-coding variants (0.2%), splice variants (12.1%), start-loss variants (0.4%), frameshift variants (20.5%), indels (1.4%), stop variants (14.1%) and missense variants (40.6%). Source data are provided as a Source Data file.

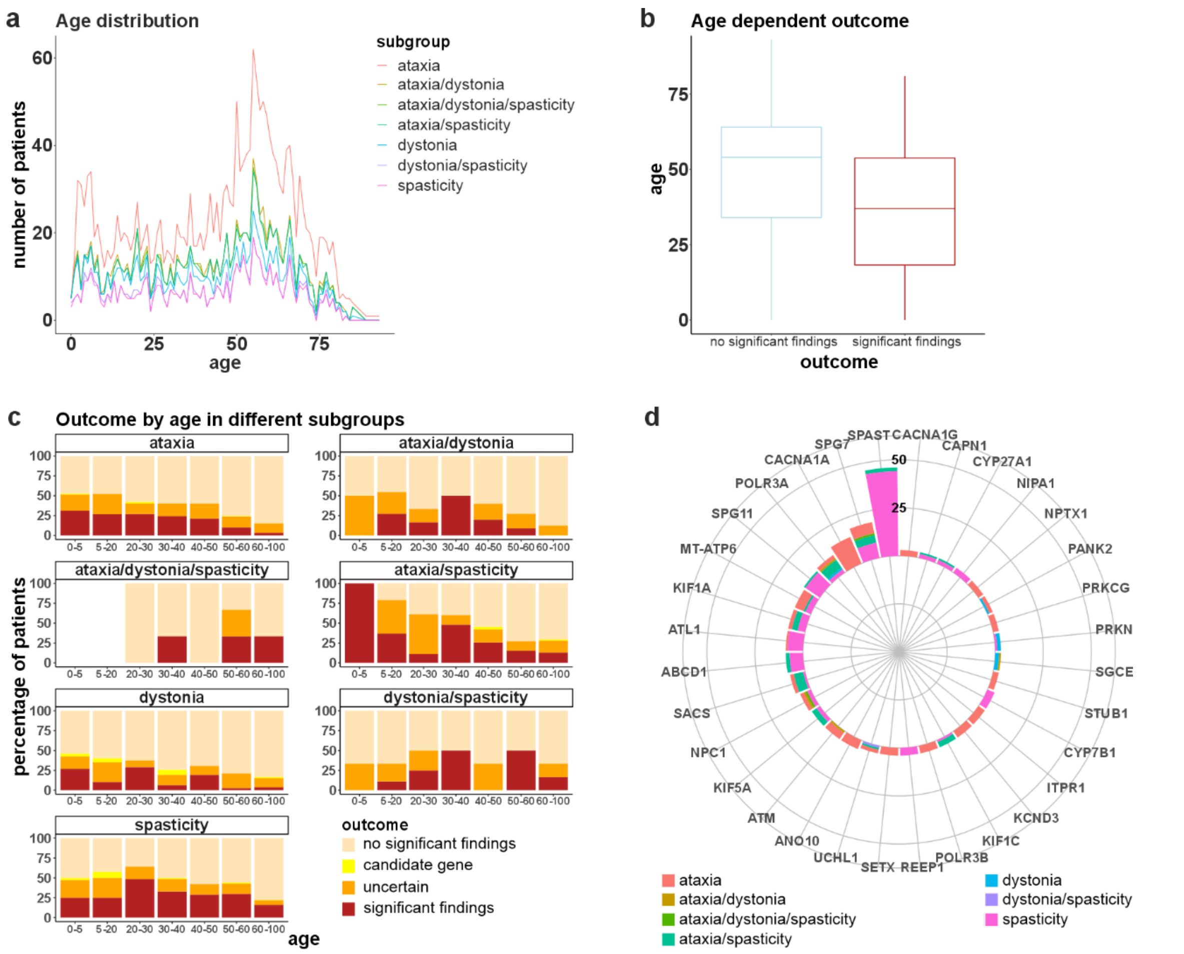

Supplementary Figure 3: Subgroups, age-dependent outcome and findings in ES cohort.

a, Age distribution of the cohort for assigned subgroups. Age refers to age at diagnosis. **b,** Median age at diagnosis was 37 y for patients with ACMG class 4/5 findings (*n* = 394) compared to 54 y for patients without findings (*n* = 1296) (Mann-Whitney U test, *P*-value < 2.2e-16, min age (no significant findings) = 0 y, min age (significant findings) = 0 y, lower quartile (no significant findings) = 34 y, lower quartile (significant findings) = 18.25 y, upper quartile (no significant findings) = 64 y, upper quartile (significant findings) = 53.75 y, max age (no significant findings) = 93 y, max age (significant findings) = 81 y).). **c,** Percentual outcome by age in different subgroups, ataxia (*n* = 899, 0-5 y, *n* = 70, significant findings 31.4%, 5-20 y, *n* = 105, significant findings 26.7%, 20-30 y, *n* = 52, significant findings 26.9%, 30-40 y, *n* = 62, significant findings 24.2%, 40-50 y, *n* = 132, significant findings 21.2%, 50-60 y, *n* = 214, significant findings 10.3%, 60-100 y, *n* = 254, significant findings 3.4%), ataxia/dystonia (*n* = 47, 0-5 y, *n* = 4, significant findings 0%, 5-20 y, *n* = 11, significant findings 27.3%, 20-30 y, *n* = 6, significant findings 16.7%, 30-40 y, *n* = 2, significant findings 50%, 40-50 y, *n* = 5, significant findings 20%, 50-60 y, *n* = 11, significant findings 9.1%, 60-100 y, *n* = 8, significant findings 0%), ataxia/dystonia/spasticity (*n* = 13, 20-30 y, *n* = 3, significant findings 0%, 30-40 y, *n* = 3, significant findings 33.3%, 40-50 y, *n* = 1, significant findings 0%, 50-60 y, *n* = 3, significant findings 33.3%, 60-100 y, *n* = 3, significant findings 33.3%), ataxia/spasticity (*n* = 215, 0-5 y, *n* = 2, significant findings 100%, 5-20 y, *n* = 19, significant findings 36.8%, 20-30 y, *n* = 18, significant findings 11.1%, 30-40 y, *n* = 25, significant findings 48%, 40-50 y, *n* = 31, significant findings 25.8%, 50-60 y, *n* = 59, significant findings 15.3%, 60-100 y, *n* = 61, significant findings 13.1%), dystonia (*n* = 265, 0-5 y, *n* = 26, significant findings 26.9%, 5-20 y, *n* = 57, significant findings 10.5%, 20-30 y, *n* = 24, significant findings 29.2%, 30-40 y, *n* = 31, significant findings 6.5%, 40-50 y, *n* = 36, significant findings 19.4%, 50-60 y, *n* = 38, significant findings 2.6%, 60-100 y, *n* = 53, significant findings 3.8%), dystonia/spasticity (*n* = 29, 0-5 y, *n* = 3, significant findings 0%, 5-20 y, *n* = 9, significant findings 11.1%, 20-30 y, *n* = 4, significant findings 25%, 30-40 y, *n* = 2, significant findings 50%, 40-50 y, *n* = 3, significant findings 0%, 50-60 y, *n* = 2, significant findings 50%, 60-100 y, *n* = 6, significant findings 16.7%), spasticity (*n* = 573, 0-5 y, *n* = 36, significant findings 25%, 5-20 y, *n* = 92, significant findings 25%, 20-30 y,

n = 64, significant findings 48.4%, 30-40 y, *n* = 64, significant findings 32.8%, 40-50 y, *n* = 69, significant findings 29%, 50-60 y, *n* = 124, significant findings 29.8%, 60-100 y, *n* = 124, significant findings 16.1%). Age refers to age at diagnosis. **d,** Genes occurring in solved cases of the different subgroups more than twice. Source data are provided as a Source Data file.

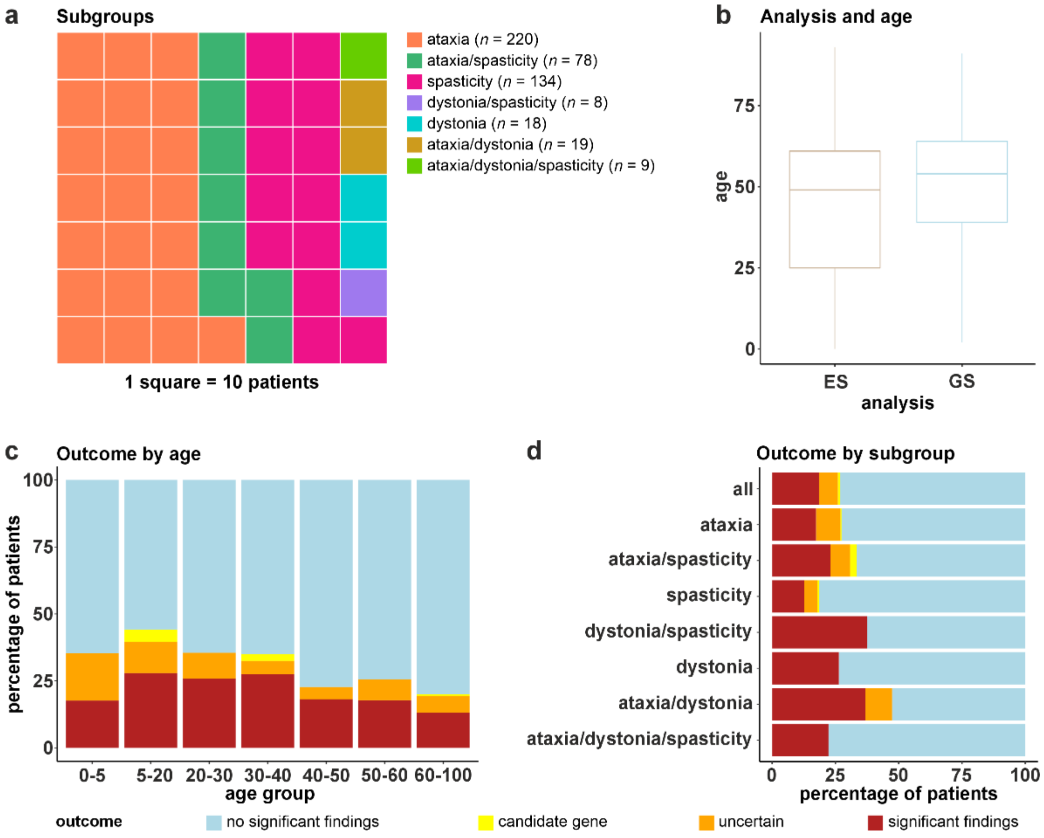

Supplementary Figure 4: Detailed description of GS cohort.

a, Subgroups in the GS cohort. **b,** Age at time of diagnosis in the ES cohort (*n* = 2041, median age 49 y, IQR 36 y, lower quartile 25 y, upper quartile 61 y, min age 0 y, max age 93 y) and GS cohort (*n* = 486, median age 54 y, IQR 25 y, median age 54 y, lower quartile 39 y, upper quartile 64 y, min age 2 y, max age 91 y). **c,** Percentual outcome by age at diagnosis in the GS cohort for the age groups 0-5 y (*n* = 17, significant findings 17.6%), 5-20 y (*n* = 43, significant findings 27.9%), 20-30 y (*n* = 31, significant findings 25.8%), 30-40 y (*n* = 40, significant findings 27.5%), 40-50 y (*n* = 66, significant findings 18.2%), 50-60 y (*n* = 129, significant findings 17.8 %), 60-100 y (*n* = 160, significant findings 13.1%). **d,** Outcome for the different subgroups in GS cohort, ataxia (*n* = 219, 17.4%), ataxia/spasticity (*n* = 78, 23.1%), spasticity (*n* = 134, 12.7%), dystonia/Spasticity (*n* = 8, 37.5%), dystonia *n* = 19, 26.3%), ataxia/dystonia (*n* = 19, 36.8%), ataxia/dystonia/spasticity (*n* = 9, 22.2%). Source data are provided as a Source Data file.

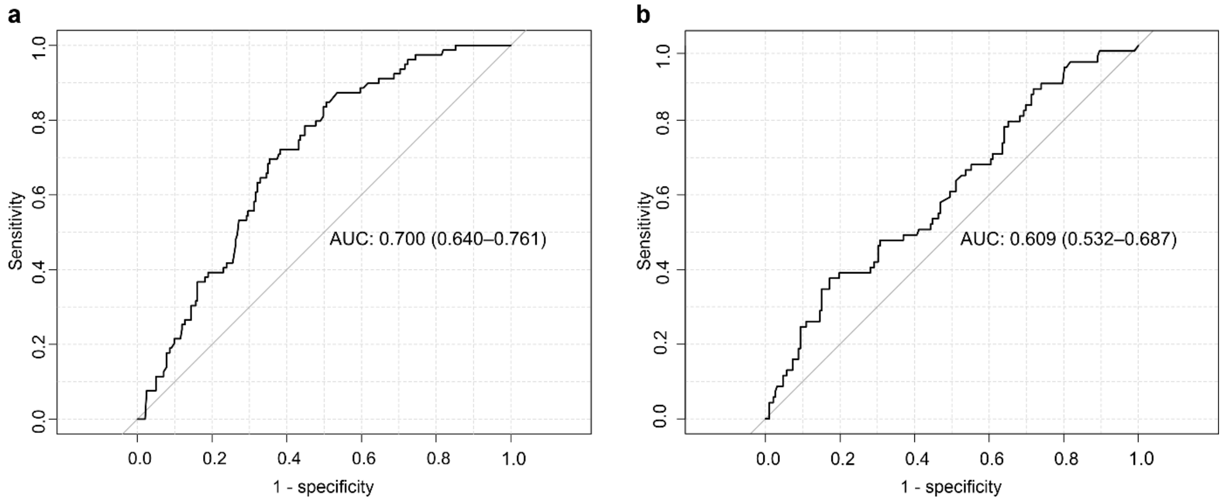

Supplementary Figure 5: Identification of factors predictive of diagnostic yield.

a, Performance of the model evaluated with the Receiver Operator Characteristics (ROC) curve derived from a training set of 1,368 cases (1,053 unsolved, 315 solved) on a test set of 322 cases (243 unsolved, 79 solved). **b,** Performance of the refitted model (given as AUC ROC) derived from the whole ES cohort of 1,690 cases (1,296 unsolved, 394 solved) on the independent genome cohort of 261 cases (192 unsolved, 69 solved). These results indicated that the trained model was able to discriminate between solved and unsolved cases irrespective of the genetic test applied. Source data are provided as a Source Data file.

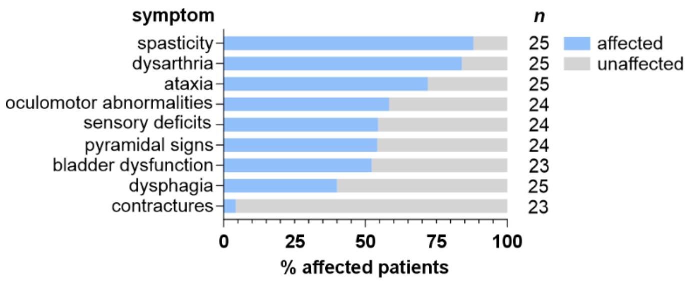

Supplementary Figure 6: Distribution of symptoms in CD99L2 patients.

Blue bars represent percentage of CD99L2-patients affected by the respective clinical feature, sorted by frequency of occurrence. Assessment of clinical signs was not available for all patients; thus, total patient numbers differ per clinical assessment. The patient numbers are displayed on the right side of the chart. Source data are provided as a Source Data file.

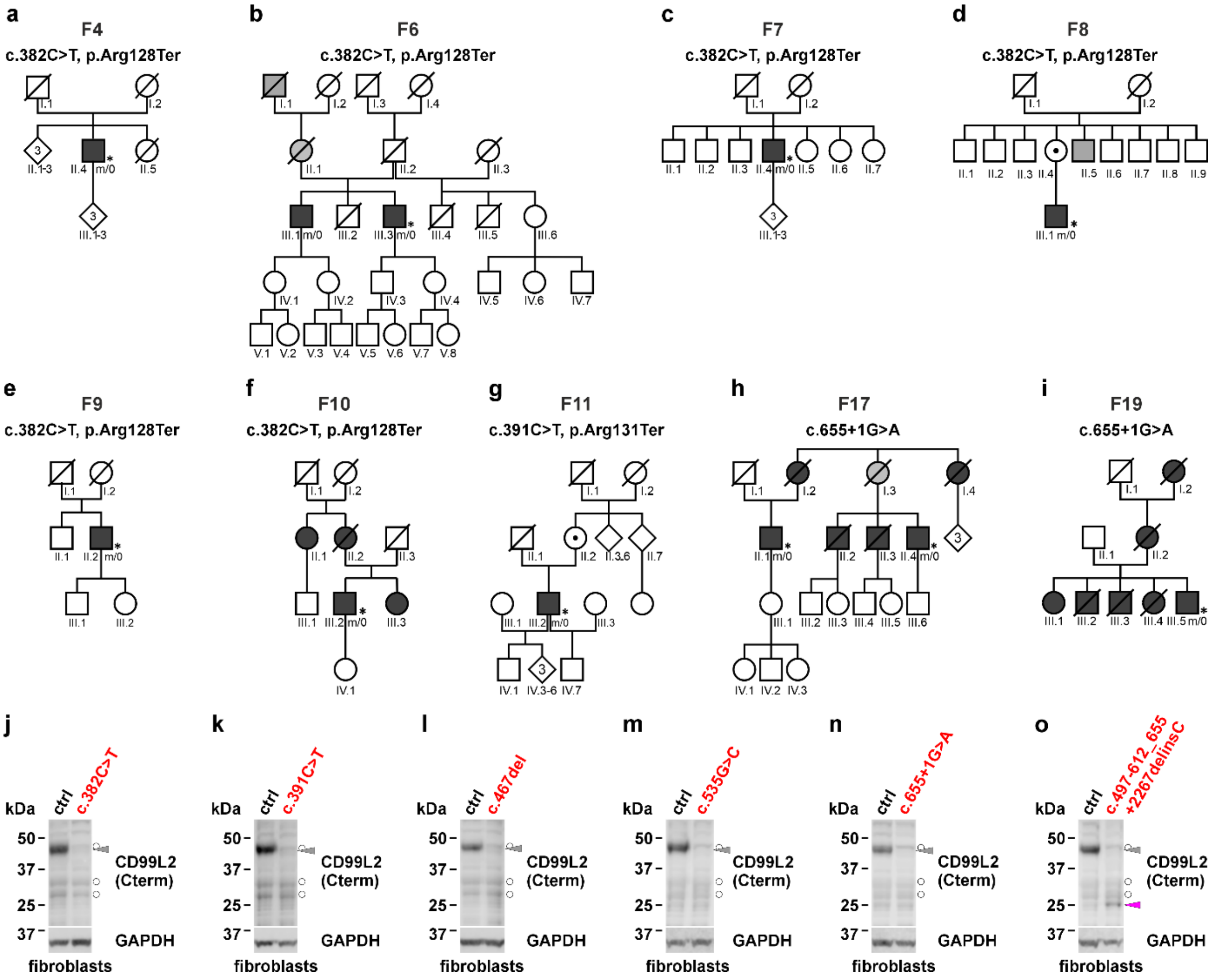

Supplementary Figure 7: Additional pedigrees of investigated families and detections of CD99L2 protein in patient-derived primary fibroblasts.

a-i, Additional pedigrees of investigated families with functionally relevant variants in *CD99L2*. Asterisks (*) indicate fully characterised patients. **j-o,** Western blotting of CD99L2 in primary fibroblasts derived from healthy control subjects (ctrl) and patients with respective variants in the *CD99L2* gene (highlighted in red script) using a C-terminally binding CD99L2 antibody. GAPDH served as a loading control. Grey arrowheads indicate the main CD99L2 band between 37°kDa and 50°kDa in the control lines. The purple arrowhead in **o** points to an internally truncated CD99L2 protein variant. White bullets mark unspecific bands. Source data are provided as a Source Data file.

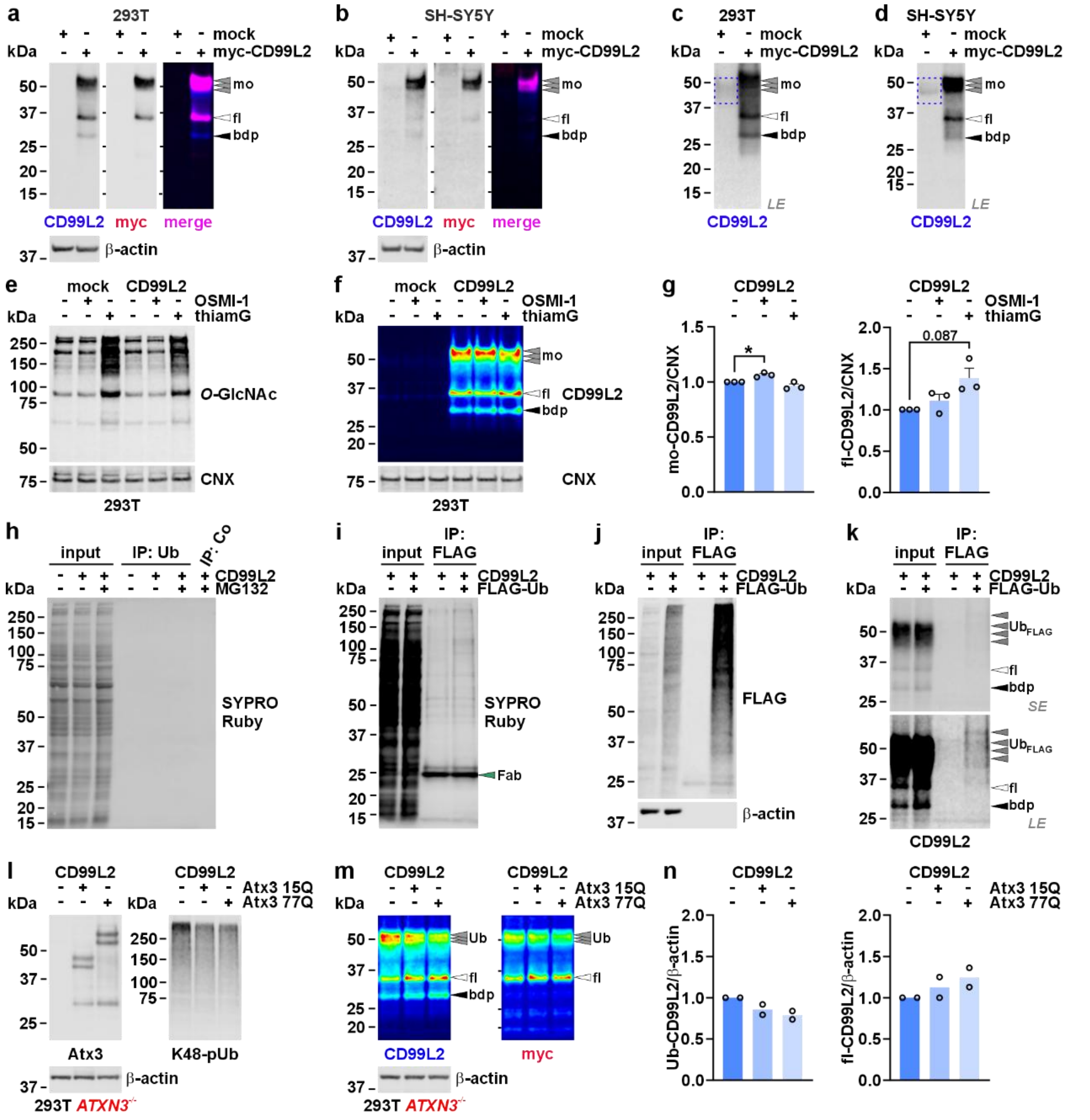

Supplementary Figure 8: Analysis of ubiquitination as a major post-translational modification of CD99L2.

a, b, Western blotting of myc-His_6-CD99L2-transfected 293T and SH-SY5Y cells using CD99L2- (blue) and myc tag (red)-specific antibodies. β-actin served as a loading control. Overlays (merge) of antibody signals are shown. Grey arrowheads indicate modified (mo) forms of CD99L2. White arrowheads show full-length (fl) CD99L2. Black arrowheads indicate a CD99L2 breakdown product (bdp). **c, d,** Long exposures (*LE*) of CD99L2-specific detections show occurrence of higher molecular, presumably modified forms (blue dashed boxes) of CD99L2 in untransfected 293T and SH-SY5Y cells. Grey arrowheads indicate modified (mo) forms of CD99L2. White arrowheads show full-length (fl) CD99L2. Black arrowheads indicate a CD99L2 breakdown product (bdp). **e, f,** Western blotting of total *O*-GlcNAc levels and CD99L2 (shown in pseudo colour) in myc-His_6-CD99L2-transfected 293T cells after 24 h treatment with *O*-GlcNAc transferase inhibitor OSMI-1 and *O*-GlcNAcase inhibitor Thiamet G (thiamG). Calnexin (CNX) served a loading control. Grey arrowheads indicate modified (mo) forms of CD99L2. White arrowheads show full-length (fl) CD99L2. Black arrowheads indicate a CD99L2 breakdown product (bdp). **g,** Densitometric analysis of modified (mo)- and full-length (fl)-CD99L2 after treatment with OSMI-1 and thiamG. Levels normalized to CNX. n = 3 biologically independent experiments. Two-tailed one sample t-test. **h,** CD99L2 ubiquitination analysis using ubiquitin-directed immunoprecipitation (IP: Ub) of myc-His_6-CD99L2 overexpressing 293T cells, treated with MG132. Total protein was stained with SYPRO Ruby. Empty control beads were used to ascertain specificity of the IP (IP: Co). **i-k,** CD99L2 ubiquitination analysis using FLAG tag-directed immunoprecipitation (IP: FLAG) of FLAG-ubiquitin (Ub_{FLAG}) and myc-His_6-CD99L2 co-overexpressing 293T cells. Total protein was stained with SYPRO Ruby (**i**), and overexpressed/immunoprecipitated ubiquitin with a FLAG-

specific antibody (**j**). A CD99L2-specific antibody was employed to detect the precipitated FLAG-ubiquitinated forms of CD99L2 (**k**). β-actin served as a loading control. Grey arrowheads indicate ubiquitinated (Ub) forms of CD99L2. White arrowheads show full-length (fl) CD99L2. Black arrowheads indicate a CD99L2 breakdown product (bdp). The green arrowhead indicates a Fab fragment band. *LE*, long exposure; *SE*, short exposure. **l, m,** Western blotting of 293T *ATXN3* knockout (293T *ATXN3*$^{-/-}$) cells co-overexpressing myc-His$_6$-CD99L2 and the deubiquitinase ataxin-3 (Atx3) with 15 or 77 glutamines (15Q and 77Q, respectively), using Atx3-, lysine-48-linked polyubiquitin (K48-pUb)-, CD99L2-, and myc tag-specific antibodies. CD99L2 and myc tag detections are shown in pseudo colour. β-actin served as a loading control. Grey arrowheads indicate FLAG-ubiquitinated (Ub_{FLAG}) forms of CD99L2. White arrowheads show full-length (fl) CD99L2. Black arrowheads indicate a CD99L2 breakdown product (bdp). **n,** Densitometric analysis of ubiquitinated and unmodified full-length CD99L2 upon Atx3 overexpression. Levels normalized to β-actin. *n* = 2 biologically independent experiments. Bars represent mean ± s.e.m. *P* values are shown in graphs. Source data are provided as a Source Data file.

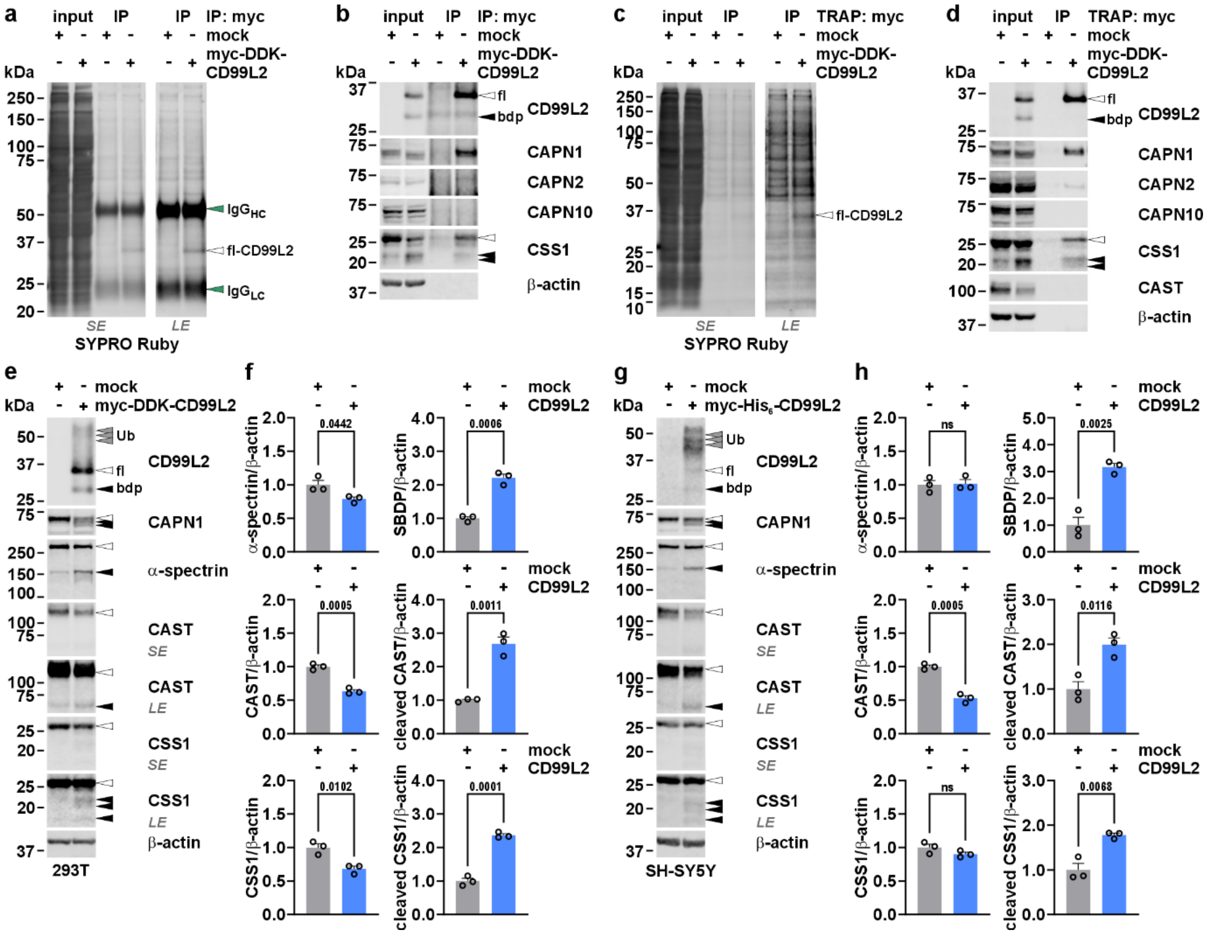

Supplementary Figure 9: CD99L2 binds and activates CAPN1.

a, b, Western blotting of myc IgG agarose-based immunoprecipitation (IP: myc) of myc-DDK-CD99L2 overexpressed in 293T cells. Total protein was stained using SYPRO Ruby. Co-immunoprecipitation of CAPN1, CAPN2, CAPN10, and CSS1 was analysed. β-actin served as a loading control. White arrowheads mark full length (fl) CD99L2 or CSS1. Black arrowheads indicate a CD99L2 breakdown product (bdp) or cleaved CSS1. Green arrowheads point to heavy chain (HC) and light chain (LC) IgG bands, respectively. **c, d,** Western blotting of myc TRAP-based immunoprecipitation (TRAP: myc) of myc-DDK-CD99L2 overexpressed in 293T cells. Total protein was stained using SYPRO Ruby. Co-immunoprecipitation of CAPN1, CAPN2, CAPN10, CAST, and CSS1 was analysed. β-actin served as a loading control. White arrowheads mark full length (fl) CD99L2 or CSS1. Black arrowheads indicate a CD99L2 breakdown product (bdp) or cleaved CSS1. **e,** Western blotting of empty vector (mock) or myc-DDK-CD99L2-transfected 293T cells using antibodies against CD99L2, CAPN1, CSS1, CAST, and α-spectrin. β-actin served as a loading control. Grey arrowheads mark ubiquitinated (Ub) CD99L2. White arrowheads indicate full-length proteins and black arrowheads the CD99L2 breakdown product (bdp) or their calpain-cleaved forms. *LE*, long exposure; *SE*, short exposure. **f,** Densitometric analysis of full-length and cleaved forms of α-spectrin, CAST, and CSS1 in CD99L2-transfected 293T cells, normalized to β-actin. SBDP, α-spectrin breakdown product. *n* = 3 biologically independent experiments. Two-tailed Student's *t*-test. **g,** Western blotting of empty vector (mock) or myc-His_6-CD99L2-transfected SH-SY5Y cells using antibodies against CD99L2, CAPN1, CSS1, CAST, and α-spectrin. β-actin served as a loading control. Grey arrowheads mark ubiquitinated (Ub) CD99L2. White arrowheads indicate full-length proteins and black arrowheads the CD99L2 breakdown product (bdp) or their calpain-cleaved forms. *LE*, long exposure; *SE*, short exposure. **h,** Densitometric analysis of full-length and cleaved forms of α-spectrin, CAST, and CSS1 in CD99L2-transfected SH-SY5Y cells, normalized to β-actin. SBDP, α-spectrin breakdown product. *n* = 3 biologically independent experiments. Two-tailed Student's *t*-test. Bars represent mean ± s.e.m. *P* values are shown in graphs. ns, not significant. Source data are provided as a Source Data file.

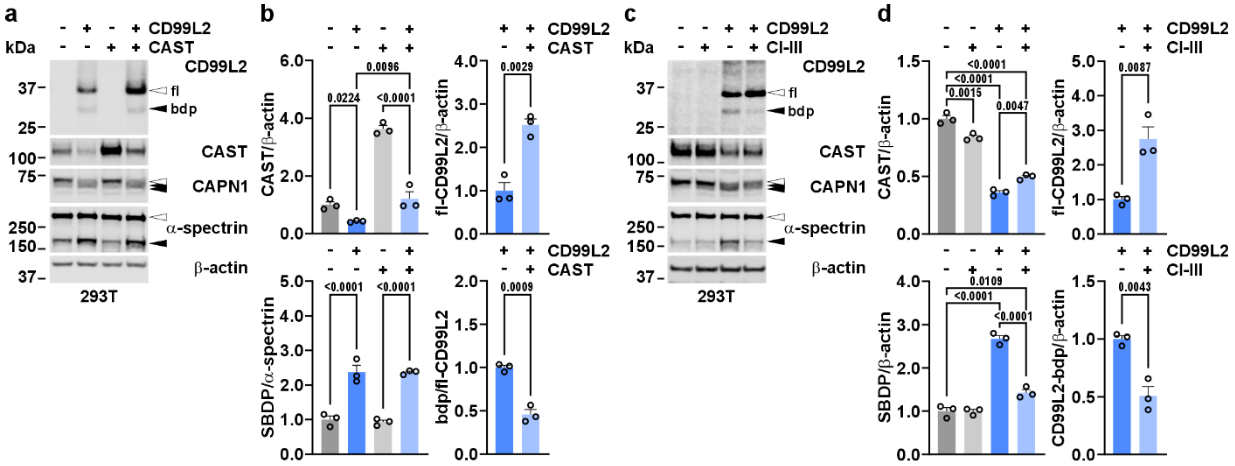

Supplementary Figure 10: CAST overexpression and pharmacological inhibitor treatment counteracts CD99L2-mediated calpain activation, while increasing CD99L2 levels.

a, Western blotting of myc-DDK-CD99L2-transfected 293T cells co-overexpressing CAST using antibodies against CD99L2, CAPN1, CAST, and α-spectrin. β-actin served as a loading control. White arrowheads show full-length (fl) proteins. Black arrowheads indicate their calpain-dependent fragments, including the CD99L2 breakdown product (bdp). **b,** Densitometric analysis of CAST, cleaved α-spectrin (α-spectrin breakdown product, SBDP), and full-length (fl) and cleaved (bdp) CD99L2, normalized to β-actin or the respective full-length protein. *n* = 3 biologically independent experiments. One-way ANOVA with Šídák's post hoc test or two-tailed Student's *t*-test. **c,** Western blotting of myc-DDK-CD99L2-transfected 293T cells after 24-h calpain inhibitor III (CI III) treatment using antibodies against CD99L2, CAPN1, CAST, and α-spectrin. β-actin served as a loading control. White arrowheads show full-length (fl) proteins. Black arrowheads indicate their calpain-dependent fragments, including the CD99L2 breakdown product (bdp). **d,** Densitometric analysis of CAST, cleaved α-spectrin (SBDP), and full-length (fl) and cleaved (bdp) CD99L2, normalized to β-actin. *n* = 3 biologically independent experiments. One-way ANOVA with Šídák's post hoc test or two-tailed Student's *t*-test. Bars represent mean ± s.e.m. *P* values are shown in graphs. Source data are provided as a Source Data file.

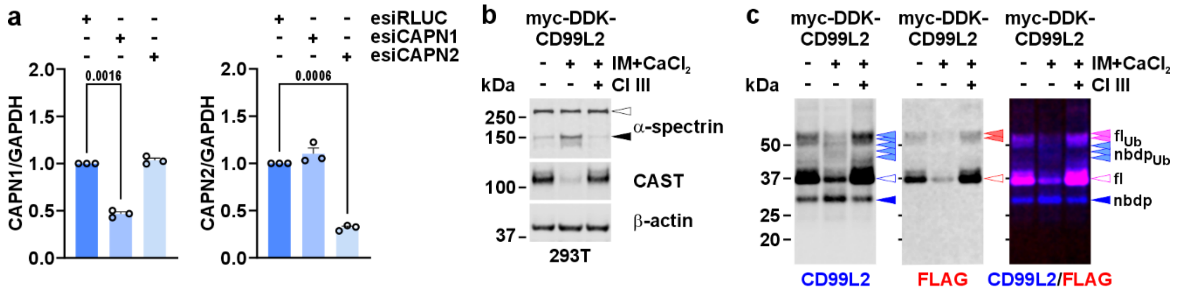

Supplementary Figure 11: CD99L2 is a target of calpain-mediated cleavage.

a, Western blotting-based densitometric analysis of CAPN1 and CAPN2 in 293T cells transfected with esiRNAs against CAPN1 (esiCAPN1), CAPN2 (esiCAPN2), or *Renilla* luciferase (esiRLUC) as a control for gene knockdown, normalized to GAPDH. *n* = 3 biologically independent experiments. Two-tailed one sample *t*-test. **b, c,** Western blotting of myc-DDK-CD99L2-transfected 293T cells after 1-h-treatment with ionomycin (IM)/$CaCl_2$ treatment for calpain activation. For specificity control, a 1-h-pre-treatment with calpain inhibitor III (CI III) was conducted. Membranes were detected with antibodies against α-spectrin and CAST (**b**), as well as N-terminal CD99L2 (blue) or the C-terminal FLAG (DDK) tag (red) (**c**). β-actin served as a loading control. The white arrowhead indicates full-length and the black arrowhead cleaved α-spectrin. Light-blue/purple/red arrowheads indicate full-length (fl_{Ub}) and/or cleaved forms (bdp_{Ub}/$nbdp_{Ub}$) of ubiquitinated CD99L2, respectively. Blue/purple/red-rimmed arrowheads indicate full-length (fl) CD99L2. Blue arrowheads indicate the N-terminal CD99L2 breakdown product (nbdp). Bars represent mean ± s.e.m. *P* values are shown in graphs. Source data are provided as a Source Data file.

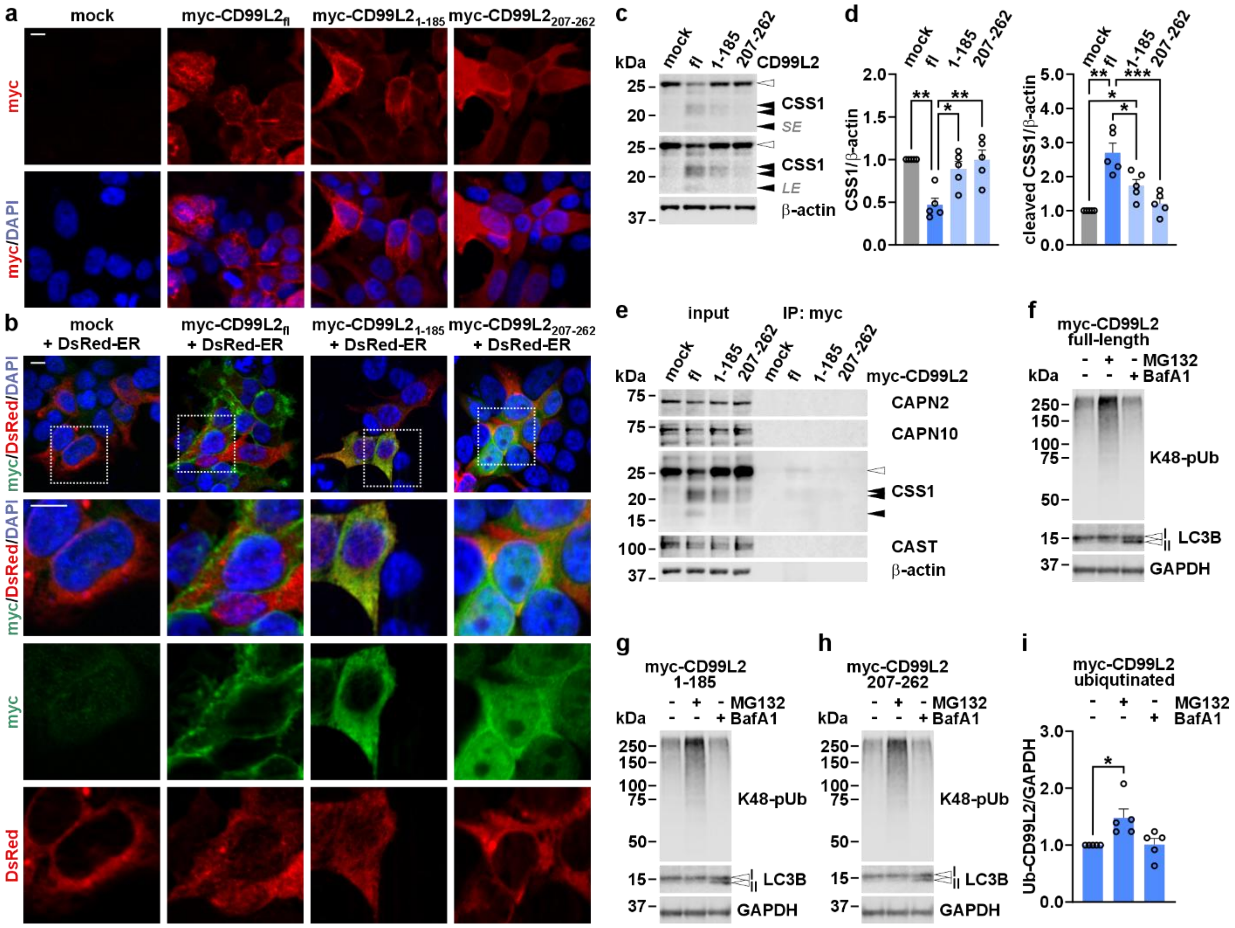

Supplementary Figure 12: Analysis of CD99L2 truncation constructs.

a, Immunofluorescence microscopy of 293T cells expressing full-length (fl)- or N- and C-terminally truncated forms ($CD99L2_{1-185}$ and $CD99L2_{207-262}$, respectively) of myc-His_6-CD99L2. CD99L2 (red) was stained using a myc tag-specific antibody. Nuclei (blue) were counterstained with DAPI. Scale bar = 10 µm. **b,** Immunofluorescence microscopy of 293T cells co-overexpressing fl- or truncated forms of myc-His_6-CD99L2 (green) along with the endoplasmic reticulum-staining DsRed-ER reporter (red). CD99L2 was stained using a myc tag-specific antibody. Nuclei (blue) were counterstained with DAPI. Lines 2-4 show 2.5x magnifications of the white-boxed field shown in the first line. Scale bar = 10 µm. **c,** Western blotting of fl-CD99L2- and truncation construct-transfected 293T cells using an anti-CSS1 antibody. β-actin served as a loading control. White arrowheads point to full-length CSS1. Black arrowheads show calpain-dependent CSS1 breakdown. *LE*, long exposure; *SE*, short exposure. **d,** Densitometric analysis of fl- and calpain cleaved CSS1, normalized to β-actin. n = 5 biologically independent experiments. Two-tailed one sample t-test, or one-way ANOVA with Tukey's post hoc test. **e,** Western blotting of co-immunoprecipitated proteins of fl-CD99L2 and its truncation constructs using antibodies against CAPN2, CAPN10, CSS1, and CAST. β-actin served as a loading control. White arrowheads point to full-length CSS1. Black arrowheads show calpain-dependent CSS1 breakdown. **f-h,** Western blotting of fl-C99L2 and truncation construct-transfected 293T cells, after 6-h-treatment with MG132 or bafilomycin A1 (BafA1) for proteasomal or autophagosomal inhibition, respectively. Membranes were detected with antibodies against K48-linked polyubiquitin (K48-pUb) or LC3B-I/II. GAPDH served as a loading control. White arrowheads indicate LC3B-I and -II. **i,** Densitometric analysis of ubiquitinated (Ub) CD99L2, normalized to GAPDH. n = 5 biologically independent experiments. Two-tailed one sample t-test. Bars represent mean ± s.e.m. P values are shown in graphs. Source data are provided as a Source Data file.

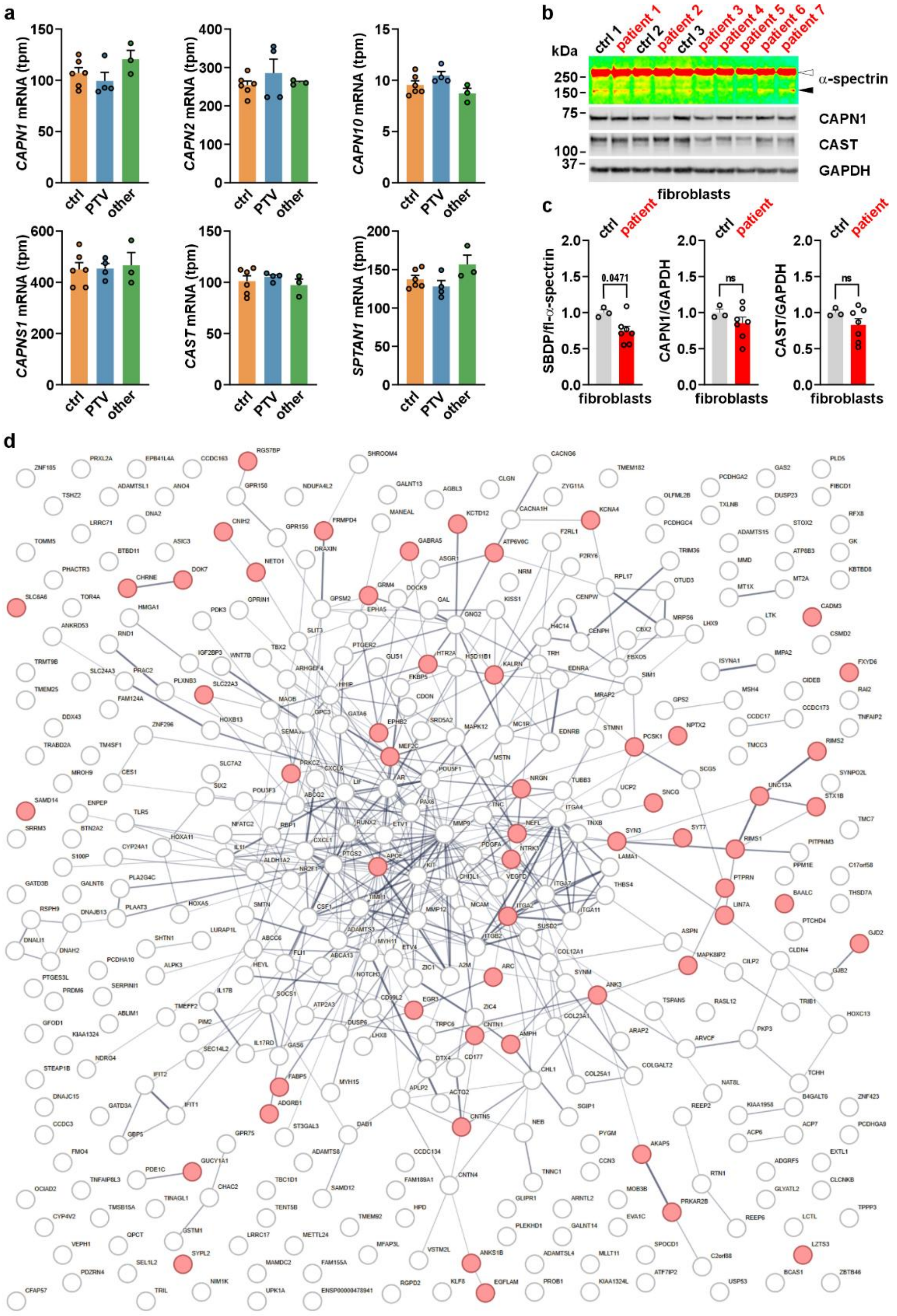

Supplementary Figure 13: Transcriptomic analysis of CD99L2 patient fibroblasts.

a, mRNA expression of calpain-related genes (*CAPN1*, *CAPN2*, *CAPN10*, *CAPNS1*, *CAST*, and *SPTAN1* (α-spectrin)) in fibroblasts of CD99L2 patients grouped in two categories: protein-truncating variants (PTV, blue, *n* = 4 cell lines) and other variants (splice-site variants and deletions, green, *n* = 3 cell lines), compared to matched healthy controls (ctrl; orange, *n* = 6 cell lines). Values are shown as transcripts per million (tpm). **b,** Western blotting of α-spectrin (shown in pseudo colour), CAPN1, and CAST in fibroblasts derived from CD99L2 patients (*n* = 7 cell lines) and matched healthy controls (ctrl; *n* = 3 cell lines). GAPDH served as a loading control. White arrowhead points to full-length α-spectrin. Black arrowhead shows the calpain-derived α-spectrin breakdown product (SBDP). **c,** Densitometric analysis of SBDP, CAPN1, and CAST in fibroblasts derived from CD99L2 patients and matched healthy controls (ctrl), normalized to β-actin or the respective full-length (fl) protein. *n* = 3 (cell lines

from 3 controls) and 7 (cell lines from 7 patients). Two-tailed Student's *t*-test. **d,** STRING-based (string-db.org) network analysis of 515 significant differentially expressed genes (DEGs) ($P \leq 0.05$; dotted horizontal line; $log_2FC \leq -0.5$ or ≥ 0.5) identified in CD99L2 patient fibroblasts compared to matched healthy controls. DEGs associated with the gene ontology term "synapse" (a total of 55 genes) are highlighted in red. Bars represent mean ± s.e.m. *P* values are shown in graphs. Source data are provided as a Source Data file.

3. Illustrative examples for the added diagnostic yield of genome sequencing

Example 1: Improved variant calling due to longer read length in genome sequencing

A 54-year-old man presented with progressive gait spasticity. In his childhood, he had frequently stumbled and reported hyperreflexia of the lower extremities. At the age of 36 years, he developed clumsiness, difficulty lifting his legs, muscle cramps in the soles of his feet (3-4 times daily), and clonus after weight bearing. His motor impairments progressed slowly until walking was only possible with walking sticks and handrail support to use stairs. He developed contractures in his hips, paraesthesia in his hands and feet, temperature insensitivity, erectile dysfunction, nocturia, and urinary retention. Neurological examination at the age of 55 years revealed a spastic gait with bilateral foot drop, spasticity of knee extension and hip adduction, paresis of hip abduction, pallhypaesthesia, and hyperreflexia (SPRS: 17/52).

Laboratory tests revealed no abnormalities in VLCFA levels and lysosomal enzyme activity, and a borderline vitamin B12 deficiency was successfully treated.

His mother and maternal grandmother were similarly affected and genetic testing of SPG4, SPG7 and SPG31 were previously unremarkable in his mother.

Initial exome sequencing and MLPA of *SPAST* revealed no pathogenic findings in the patient. Unexpectedly, subsequent genome sequencing revealed a known heterozygous pathogenic frameshift *SPAST* variant (ENST00000315285: c.67_85dup, p.Leu29Glnfs*25) (Example 1 Fig. 1). This variant was validated in the patient and confirmed in the affected mother by Sanger sequencing (Example 1 Fig. 2). Manual inspection of the respective positions in the exome and genome datasets suggested that this variant has not been called despite superior coverage (95X) in the exome experiment possible due to the slightly shorter read length of 125 bp, with only 18/95 reads correctly indicating the variant. In the genome data generated as 150 bp reads the change was correctly called albeit with rather low quality (QUAL = 133; DP = 40; QD = 3.33; AF = 0.28; MQM = 60; SAP = 13; ABP = 21). This observation is in line with a slightly longer read length of genome sequencing protocols supporting the detection of small insertions and deletions due to improved calling with respective algorithms.

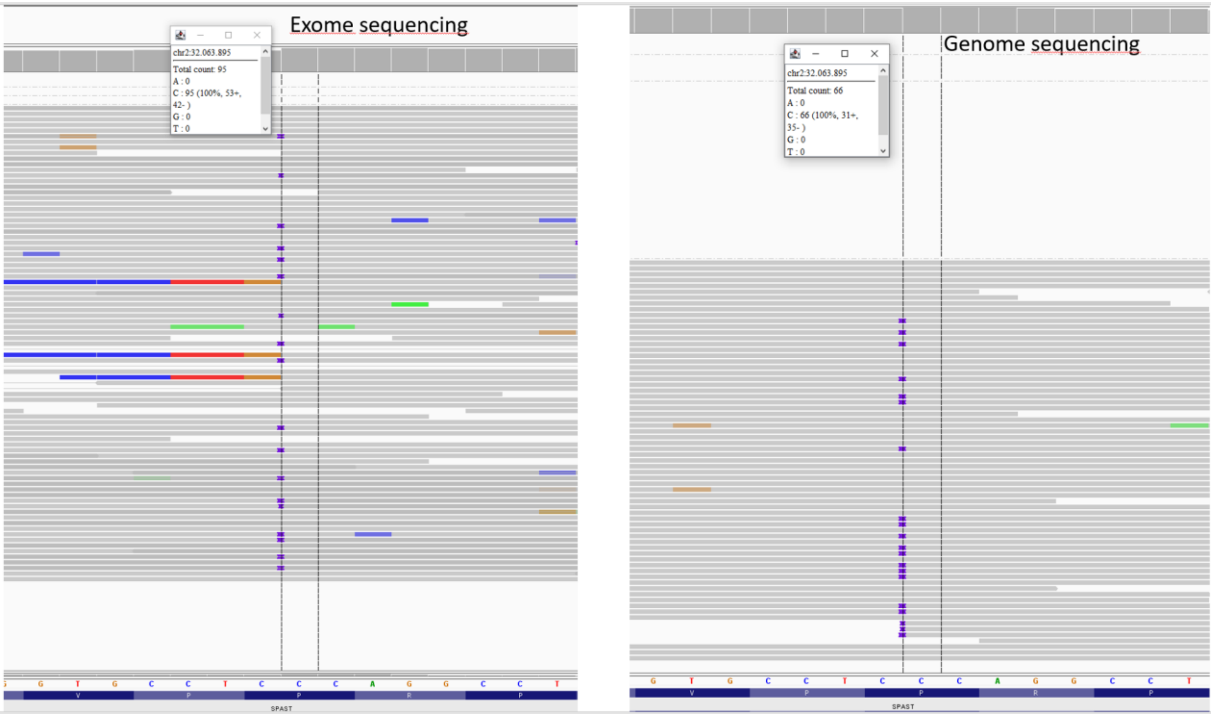

Example 1 Figure 1: Genome sequencing (right panel) revealed a heterozygous pathogenic 19 bp duplication in *SPAST* that has been missed despite higher coverage in a previous exome analysis.

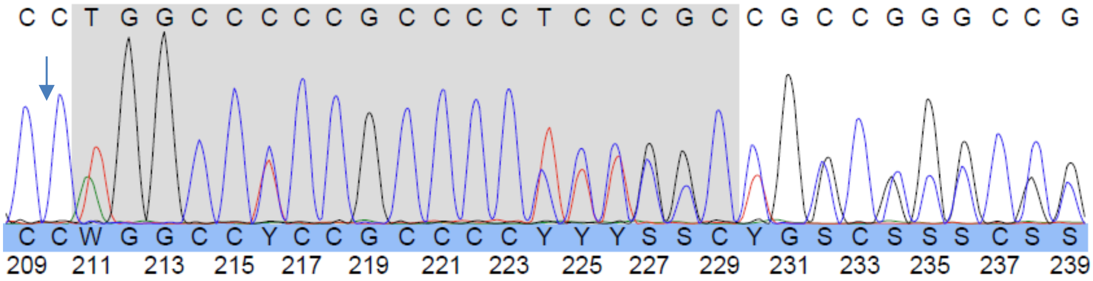

Example 1 Figure 2: The identified *SPAST* variant was subsequently confirmed in the index patient using Sanger sequencing.

Example 2: Second allele in noncoding first exon missed by exome sequencing in a recessive disease

A 42-year-old woman presented with early-onset ataxia, mild intellectual disability, and severe myopathy. In infancy, she had developmental delay in cognitive and motor functions, scoliosis, and dysarthria. Bilateral cataracts were surgically removed at age 14 and scoliosis was surgically treated at age 17 when she began using a walker. She developed marked paraparesis, which eventually extended to the upper extremities and resulted in impaired fine motor skills.

Clinical examination revealed discrete dysmorphic signs (narrow face and nose and convergent strabismus), large amplitude rectangular twitching, high-grade paresis of the upper and lower extremities with atrophy, cerebellar dysarthria, marked distal edema of the legs and partial loss of toenails. Standing and walking were not possible even with support.

Lysosomal enzymes were normal, brain MRI showed cerebellar and vermis atrophy, as well as an enlargement of the 4th ventricle and superior cerebellar and pontocerebellar cisterns. Electrophysiology showed signs of sensor-motor polyneuropathy. Other family members were not affected. Due to the characteristic triad of tetraparesis of myopathic origin, bilateral cataract and ataxia at a young age as well as additional mild dysmorphic signs, scoliosis and mild mental retardation, Marinesco-Sjörgren syndrome was clinically suspected.

However, exome sequencing revealed only a single heterozygous pathogenic frameshift variant in *SIL1* (ENST00000394817.7: c.947dup, p.Arg317Glufs*35). Subsequent genome sequencing re-identified the heterozygous frameshift variant but in addition detected a heterozygous splice site variant affecting the first noncoding exon which had not been covered by the exome enrichment. A subsequent RNA sequencing analysis in patient-derived fibroblast showed the frameshift variant in an apparently homozygous state, indicating together with significantly reduced *SIL1* expression levels a nonsense-mediated decay of the allele carrying the splice site alteration (Example 2 Fig.1).

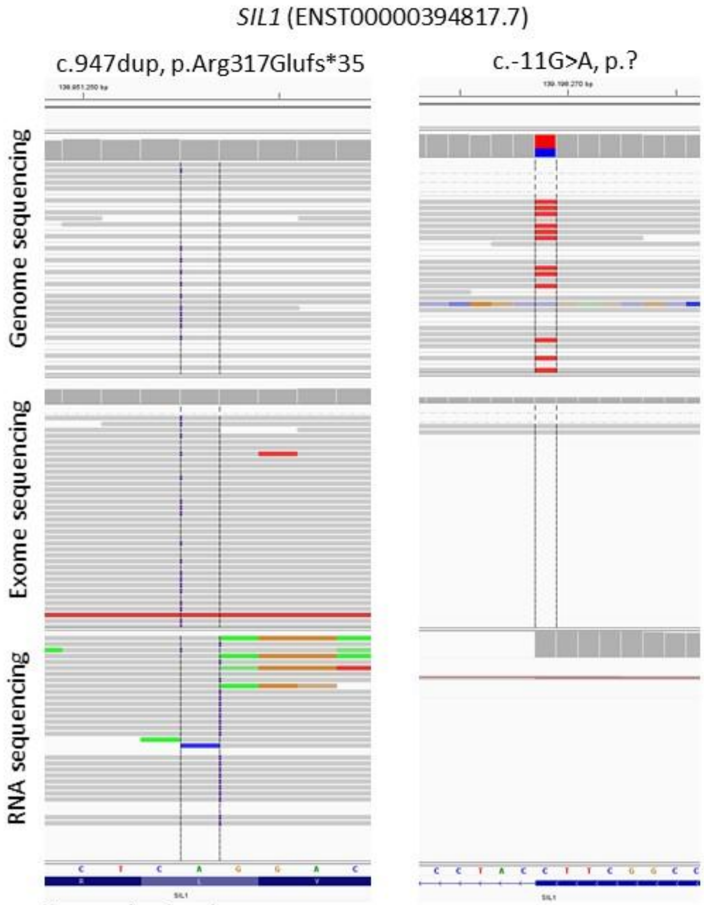

Expression levels

	gene_id	gene_name	gene_biotype	raw	tpm	cohort_mean	log2fc	zscore	pval
1	ENSG00000036257	CUL3	protein_coding	943	7.60	28.39	-1.72	-3.981	0.000
2	ENSG00000120725	SIL1	protein_coding	462	10.77	58.65	-2.25	-3.954	0.000
3	ENSG00000158623	COPG2	protein_coding	273	6.12	35.22	-2.24	-3.736	0.000

Example 2 Figure 1: Visualization of the generated genome, exome, and RNA datasets by Integrative Genomics Viewer (IGV) showing the coverage of the positions of the two alterations in the respective experiments. The splice variant was only detected by genome sequencing with the apparently homozygous state of the frameshift variant in the RNA sequences suggesting a nonsense-mediated decay of the allele carrying the splice variant.

Example 3: Second allele with copy-neutral inversion in recessive disease

A 30-year-old man presented with suspected chorea-acanthocytosis associated with motor symptoms, epilepsy, and myopathy. A pathogenic heterozygous *VPS13A* frameshift variant (c.6059del, p.Pro2020Leufs*9) and variant of unknown significance (c.1596-18C>T, p.?) had previously been detected in cis (verified by segregation). A loss of chorein was confirmed by western blot. In addition to the known frameshift variant, genome sequencing of the index patient revealed an inversion of exons 36 to 40 in *VPS13A*. The breakpoints of the inversions are located within the intronic regions and were therefore not detected by the previous exome analysis. The biallelic localization of the variants was confirmed by carrier testing of the parents and both changes were confirmed in the similarly affected brother.

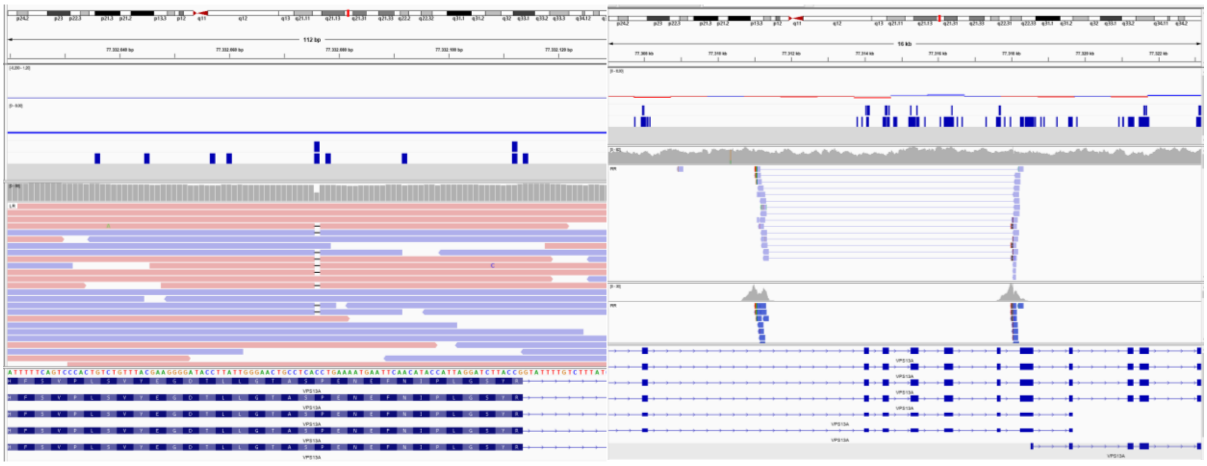

Example 3 Figure 1: Visualization of the generated genome datasets by Integrative Genomics Viewer (IGV) showing the coverage of the heterozygous *VPS13A* frameshift variant as well as the heterozygous inversion involving exons 36 to 40, resulting in a disruption of the open reading frame.

Example 4: Homozygous copy-neutral inversion in recessive disease missed by exome analysis

A 49-year-old woman who presented with a movement disorder that manifested primarily as a progressive hypokinetic-rigid syndrome and evidence of acanthocytes in the blood smear. The loss of chorein was confirmed by western blot analysis. No other family members were affected. Targeted genetic testing of *VPS13A* and other genes associated with chorea-like movement disorders (ADCY5, FTL, NKX2-1, NLRP3, PRNP, PRRT2, RNF216) was unremarkable. Genome sequencing revealed a homozygous inversion of exons 36-40 with the parents being heterozygous carriers.

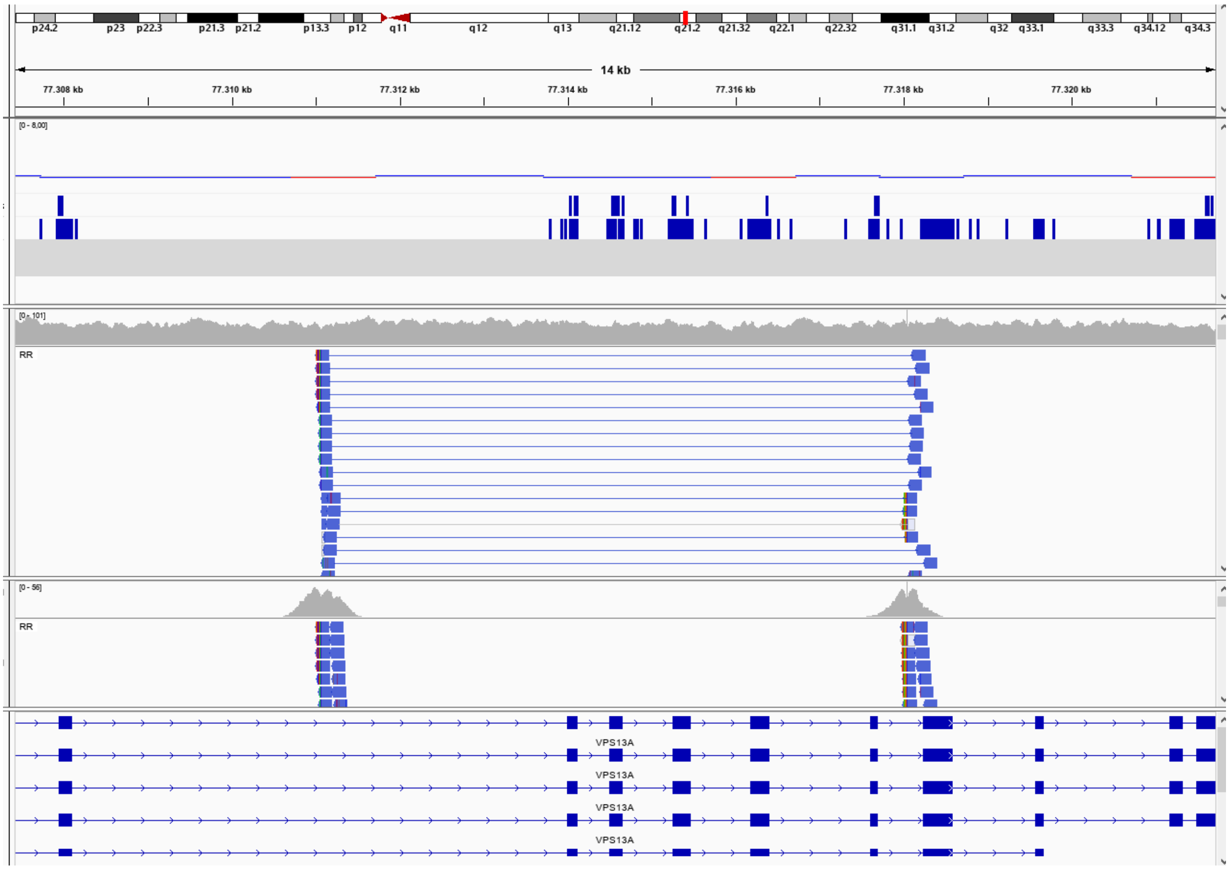

Example 4 Figure 1: Visualization of the generated genome dataset by Integrative Genomics Viewer (IGV) showing a homozygous inversion involving exons 36 to 40, resulting in a disruption of the open reading frame.

Example 5: Detection of a repeat expansion in FXN via GS in an atypical case of Friedreich ataxia

A 64-year-old woman presented with complicated spastic paraplegia with cerebellar signs. At the age of 52, she noticed stiffness in her leg muscles after walking. Her symptoms progressed and she developed hoarse speech, dysphagia, and fine motor coordination problems as well as urinary incontinence. She was confined to a wheelchair at the age of 63.

Clinical examination showed a wide-based ataxic gait, cerebellar dysarthria, hyperreflexia, particularly in the lower limbs, pallhypaesthesia, and intention tremor. Laboratory tests revealed normal VLCFA levels and normal lysosomal enzyme function.

There was a family history of an affected brother who, at the age of 57, had a severe speech disorder together with a gait disorder.

DNA fragment analysis for SCA 1,2,3,6,7 and 17 and C9orf72 as well as exome sequencing were initially performed, which revealed a heterozygous missense variant of unknown significance in *ABCD1* (ENST00000218104.6: c.1084G>T, p.Ala362Ser3). Subsequent genome sequencing and including a repeat analysis using ExpansionHunter indicated a homozygous GAA expansion of both alleles of *FXN* of about 83 repeats. No spanning reads were reported, suggesting a larger expansion. The results were confirmed by conventional fragment analysis (Example 5 Fig. 1).

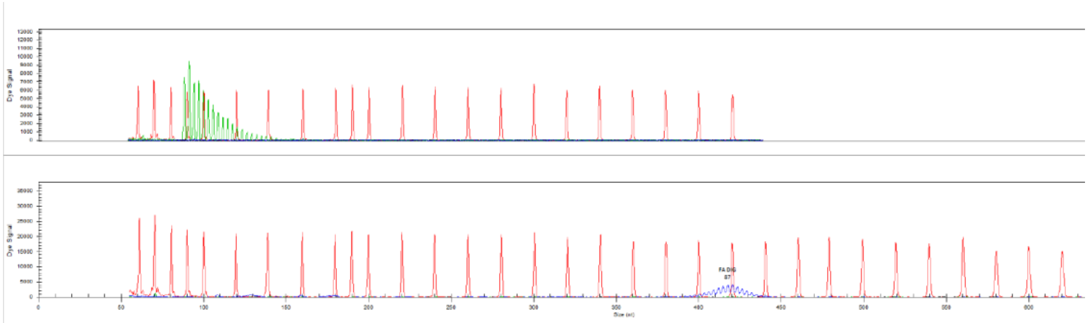

Example 5 Figure 1: Fragment length analysis indicating a biallelic expansion in *FXN* (GAA-repeats: ~96/~1140). Upper panel control, lower panel affected individual.

4. Case reports *CD99L2* families

Family 1, individual II.1 [c.51del, p.Thr18ProfsTer19]

This 79-year-old male of German descent presented with an 11-year history of slowly progressive gait disorder with repeated falls, slurred speech, and mild dysphagia. Family history was negative for gait disturbances. His sister developed a non-specified cognitive decline at age 73 years.

He had normal motor and cognitive development. First symptoms appeared at the age of 68. He noticed gait unsteadiness, which worsened on uneven surfaces, in the dark and when performing multitasking activities. At the age of 78, he used walking sticks outside the home. He described the sensation of incomplete bladder emptying without sonographic evidence of residual urine.

Neurological examination revealed afferent ataxia of the limbs, gait and stance with absent vibratory sensation and impaired joint position sensation of the lower limbs and absent ASR, but also cerebellar involvement manifesting as cerebellar dysarthria, with no clear oculomotor abnormalities. In addition, the upper motor neuron was affected with mild paraparesis, brisk stretch reflexes and positive Babinski sign.

Neurophysiology revealed axonal sensory-motor neuropathy. Motor evoked potentials showed prolonged central motor conduction time. MRI of the brain showed white matter lesions that were most likely of microangiopathic origin. MRI of the spine showed degenerative changes and a herniated disc L4/L5. The basic parameters in the CSF were normal, as were the neurofilament light values.

Family 2, individual III.1 [deletion exons 9-11]

This 55-year-old male presented with a 38-year history of slowly progressive gait disorder. He reported first problems with his right leg around the age of 17 years, and his condition reportedly worsened significantly over the last 5 years. He did not require a walking aid. The maternal family history was negative for neurological disorders.

Neurological examination showed a spinocerebellar syndrome including a mild dysarthria, moderate paresis of both lower extremities with increased reflexes, positive Babinski sign on the right side and dysmetria mainly of the right side, including the right arm.

MRI was normal, with no spinal stenosis or significant cerebellar abnormalities. Electrophysiological studies did not reveal any evidence of myopathy. Slightly elevated protein levels were observed in the CSF.

Family 3, individual III.1 [c.281_282del, p.Arg94MetfsTer24]

This 60-year-old patient of German descent presented with a 9-year history of slowly deteriorating gait and slurred pronunciation. While his walking distance is not limited, coordination activities such as cycling or dancing are no longer possible. Three years ago, he developed a subtle irregular head tremor. There was no comparable clinical phenotype in his family.

Neurological examination revealed a spastic-ataxic gait pattern with brisk reflexes of the lower limbs and additional Babinski signs. A saccadic gaze sequence with restoring saccades and a mild dysarthrophonia were obvious. The finger-nose and especially the knee-heel test were clearly dysmetric consistent with cerebellar ataxia. The Scale of the Assessment and Rating of Ataxia (SARA) score was 20/40. On the SPRS scale 7/52 points were scored.

Dedicated neuropsychological testing showed no cognitive abnormalities. MRI of the brain showed no significant pathology or atrophy.

Family 4, individual II.4 [c.382C>T, p.Arg128Ter]

The patient developed progressive balance problems at age 41. At age 49, his first examination showed a spastic ataxic phenotype with gait ataxia, slow and dysarthric speech, appendicular ataxia with dysmetria, and mild action tremor (SARA: 13.5 points), as well as mild spasticity and hyperreflexia in the legs. A diagnostic work-up including brain imaging, nerve conduction studies, CSF and laboratory testing, and genetic testing for SCA1, 2, 3, 6, 7, and 17 was unremarkable. The patient presented again at age 62, after developing progressive asymmetric action tremor that interfered with his activities of daily living. Spastic ataxia had only been mildly progressive

over the years (SARA: 14 points, intermittent use of walker, hyperreflexia in arms), but was now also due to sensory ataxia (severely impaired vibration sense and Rombergism).

Family 5, individual III.1 [c.382C>T, p.Arg128Ter]

This 49-year-old male developed a slowly progressive cerebellar ataxia and spasticity of lower legs at the age of 39 years. He first presented in our outpatient clinic at the age of 43 years. Clinically a mild spastic paraparesis with positive Babinski sign, bulbar dysarthria, saccadic pursuit and mainly gait-ataxia was determined. Later, he developed limb ataxia and clinical signs of neuropathy. Comorbidities were diabetes mellitus, hypothyroidism and adiposity. He had a focal hepatic lesion and a neoplasm of the bladder; biopsies performed were inconclusive in both cases. Family history revealed that the grandfather on the mother's side had gait problems when he was older. Magnetic resonance imaging (MRI) of the brain showed atrophy of the cerebellum and a singular T_2-wheigted lesion periventricular left. Spinal MRI revealed normal results. Although there were clinical signs for neuropathy (decreased vibration sense and numbness of his feet's) the nerve conduction study showed none. Motor evoked potentials showed a central affection of pyramidal tracts to all four extremities and sensory evoked potentials were deducted with prolonged latency. Examination of the spinal fluid excluded an infectious or autoimmune cause of the disease. Prior genetic testing had ruled out spinocerebellar ataxia type 1-3, 6, 7 and 17.

Family 6, individual III.1 and III.3 [c.382C>T, p.Arg128Ter]

This 71-year-old male (III.1) presented with spastic paraplegia with first symptoms being recognized at the age of 47 years. He has started to use a walking aid at the age of 61 years. Furthermore, he developed dysarthria and dysphagia. Cognitive impairment was absent. His two daughters and four grandchildren are all without symptoms.

His older brother (III.3) is now aged 74 years and presented with similar clinical features. Age of onset was 50 years and he needed a walking aid from the age of 62 years on. Cognition was normal. He developed urinary urgency. Examination at age 63 years showed spastic paraplegia, upper limb ataxia and a dystonic head and arm tremor. His two children and four grandchildren are clinically unaffected.

A third brother died at the age of 49 years from cancer. Their mother died aged 85 years and was said to move in a ''stiffed'' manner, and her father (grandfather of the index) was also very stiff.

Family 7, individual II.4 [c.382C>T, p.Arg128Ter]

This 75-year-old male presented with a slowly progressive cerebellar ataxia that started at the age of 32 years with gait disturbances. There was dysphagia, and he complained about some urinary urgency. Physical examination at age 74 years showed a spastic ataxic gait without any other pyramidal signs, some hypometric and slow saccadic eye movements and mixed dysarthria. Brain MRI showed mild cerebellar atrophy. Family history was negative for ataxia or spastic paraplegia.

Family 8, individual III.1 [c.382C>T, p.Arg128Ter]

This 51-years-old male presented with slowly progressive walking difficulties evolving from the age 42 years on. Over the years, speech problems and mild cognitive changes emerged. He has a past medical history of Crohn's disease, depression, mild obstructive sleep apnea, and eczema.

Neurological examination at the age of 50 years showed cerebellar dysarthria, mild fluency deficits, mild gaze-evoked nystagmus and hypermetric saccades, mildly increased tone of lower limbs, appendicular ataxia, brisk tendon reflexes with an equivocal extensor plantar response on the right side, and an ataxic and somewhat stiff gait. An MRI brain scan (at age 49 years) scan did not show cerebellar atrophy. Investigations into acquired and known genetic causes provided normal results, the latter including testing for CAG repeat expansions in SCA1-2-3-6-7-17 and exome sequencing targeting an extensive set of known genes associated with movement disorders. His family history revealed an asymptomatic mother (a proven carrier of the *CD99L2* variant), but her brother was said to have possibly similar gait and speech difficulties; he was not available for clinical or genetic evaluations. Six other male siblings of the mother were reportedly healthy.

Family 9, individual II.2 [c.382C>T, p.Arg128Ter]

This 66-year-old male first developed a gait instability and vertigo at the age of 55 years. Clinically, we determined a cerebellar syndrome and a polyneuropathy with sensory ataxia. He had no other pre-existing conditions. Family history revealed a 75-year-old brother who was diagnosed with Parkinson's disease at the age of 58. The patient's mother had a walking disability, which had been attributed to food poisoning. She was confined to a wheelchair and died at the age of 97.

Brain MRI and MRI of the cervical spine showed no morphological abnormalities, in particular not of the cervical myelon and cerebellum.

Electroneurography revealed a predominantly axonal, beginning also demyelinating, sensorimotor polyneuropathy of the multiplex distribution type. Correlating with this, electromyography revealed a chronic neurogenic damage pattern with emphasis on the proximal limb muscles. SEP and MEP additionally indicated a central and peripheral conduction disorder of somatosensory as well as motor pathways.

Prior genetic studies included fragment length analyses for repeat expansions associated with fragile X-associated tremor-ataxia, SCA1, 2, 3, 6, 7, 8, 10, 12 and 17, as well as panel sequencing, all with unremarkable results.

Family 10, individual III.2 [c.382C>T, p.Arg128Ter]

This 62-year-old male reported presented with a slowly progressive spastic gait disorder developing from the age 58 on. Clinical examination revealed a SPRS with 7/52 points with brisk reflexes of the lower limbs, a mild increase of the muscle tone of the adductor muscles of the lower limbs (1-2 points on the modified Ashworth scale) without pyramidal signs or any associate neurological signs. Brain and spinal MRI imaging at the age of 62 were unremarkable, as were the nerve conduction of the upper extremities and lower limbs. The motor evoked potential revealed a prolonged central motor conduction time to the left lower limb. The sensory evoked potentials were prolonged from both the upper extremities and lower limbs. Laboratory chemistry revealed no abnormalities in either serum or cerebrospinal fluid including the neurofilament light and heavy chain.

Family 11, individual III.2 [c.391C>T, p.Arg131Ter]

This 64-year-old male of German descent presented with a nine-year history of progressive spastic ataxic gait disorder but with unlimited maximum gait distance (up to 15 km possible) despite an increased effort. Additional reported symptoms were slowing of speech (not self-recognized but by relatives and friends), dysphagia (choking on own saliva), a urinary urge with occasional incontinence and erectile dysfunction. The clinical examination showed a spastic paraparesis with a wide-based arrhythmic spastic gait pattern and additional ataxia of the extremities on the left side. Pseudobulbar dysarthria. Reflexes were normal, Babinski sign was negative. The SARA score was 5,5 points.

Family 12, individual III.3 [c.391C>T, p.Arg131Ter]

This 61-year-old Caucasian male of nonconsanguineous parents developed slowly progressive unsteadiness of gait in his mid-fifties. In the course of the disease he also developed difficulties swallowing and mild dysarthria. After longer periods of rest his legs felt stiff. There is mild pollakisuria. On neurological examination he showed a cerebellar syndrome with mild dysarthria, mild ataxia of stance and gait and mild limb ataxia. Brain MRI scan was normal with no signs of cerebellar atrophy. Sensory and motor evoked potentials were abnormal. Nerve conduction studies did not show sensory neuropathy; nerve conduction of the tibial nerves was slow. Family history was negative.

Family 13, individuals III.1-4 and II.2-3 [c.467del, p.Gly156ValfsTer71]

This family consists of two affected brothers, and three female carriers, two of them with putative minor neurological signs.

The 68-year-old male index patient (III.2) presented with the complaint of insidiously progressive gait disturbance and dysarthria starting at age 46. Neurological examination revealed lower leg spasticity, decreased vibration

sense of the lower limbs and cerebellar signs of saccadic ocular pursuit, dysarthria and limb ataxia. He also had restless legs syndrome which responded well to dopaminergic therapy. CSF analysis and MRI of the brain were normal, whereas spinal MRI revealed non-compressive spinal cervical and lumbar stenosis. Symptoms progressed slowly over time and at the age of 68, gait was slowed but he did not need a walking aid, and maximum gait distance was not limited. There were frequent falls and his ability to write was compromised. The diagnosis of an adult-onset spinocerebellar ataxia was made.
Similarly, a 72-year-old brother (III.3) showed a gait disorder starting at the age of 47, with moderate lower limb spasticity and gait and limb ataxia with a marked cerebellar tremor of the upper extremities. This patient's brain and cervical MRI were normal and he had no restless legs syndrome. At the age of 72, his maximum gait distance was 2 kilometers, there were frequent falls and he was significantly impaired in writing and eating due to ataxia. They had one unaffected brother (III.1) and one unaffected sister (III.4). Their mother (II.2) showed mild head tremor, suspected to be of dystonic etiology. The mother's sister (II.3) showed mild leg spasticity from the age of 70, but there were no signs of ataxia or other neurological findings.

Family 14, individual III.2 [deletion exons 8, 9]

This 57-year-old Caucasian male developed a slowly progressive gait and speech disorder at the age of 50. He had a medical history of hypertension, dyslipidemia and gastro-esophageal reflux-disease. Furthermore, he experienced bilateral mastectomy because of a benign gynecomastia and a testosterone deficiency, probably after sport-associated steroid abuse in the youth. Family history concerning neurological symptoms, especially gait disturbances was inconspicuous.
Clinical examination revealed a moderate spasticity and mild fasciculation of the lower limbs, brisk tendon reflexes on upper and lower limbs, pyramidal signs and a slight pseudobulbar dysarthria. There were no psychiatric symptoms, cognitive decline, involvement of the visual system or bladder and bowel dysfunction. Spastic paraplegia rating scale (SPRS) at baseline was 12 points.
Laboratory tests including blood and cerebrospinal fluid analyses were unremarkable. MRI of the brain showed single, unspecific white matter lesions. MRI of the myelon did not reveal any abnormalities. Visual evoked potentials and sensory potentials of the upper limbs were normal. The examination of lower limbs sensible evoked potentials, motor evoked potentials and neurography of upper and lower limbs was not tolerated by the patient.

Family 15, individual II.2 [deletion exons 8, 9]

This 72-year-old male of German descent presented with a 16-year history of slowly deteriorating gait. His family history was negative for gait disorders or consanguinity.
He reported normal motor development and no cognitive impairment. His first symptoms at the age of 56 years comprised of the progressive feeling of stiffness and reduced walking speed. At the age of 72 years, he was still unlimited in his walking distance and not using any walking aid or antispastic medication. Vegetative symptoms were absent.
Neurological examination at the age of 72 years revealed a mild pyramidal syndrome with lower limb predominant spastic tetraparesis, brisk stretch reflexes of the upper and lower limbs, a positive Trömner and Babinski sign and bilateral sustained ankle clonus. Sensory examination was intact. Incoordination of the left hand was noted but could be attributed to cerebral infarction in the area of the left anterior inferior cerebellar artery. Additionally, a "no-no" head tremor was present.
Routine CSF examination was normal. MRI of the brain and cervical spine at age 66 years did not show relevant atrophy, corresponding to the late onset and slow progression, but old ischaemic lesions in the left posterior middle cerebral artery territory and the left cerebellum.
Besides the cerebellar ischemia, he suffered from arterial hypertension and chronic obstructive lung disease.

Family 16, individual II.2 [c.535G>C, p.Gly179Arg]

This 62-year-old male of German descent presented with a seven-year progressive spastic gait disorder with afferent ataxia and a maximum gait distance of 200 m using walking sticks. Additional neurological findings

included dysarthria, dysphagia, urinary voiding, and incontinence. The neurological examination showed a lower limb dominant tetraspasticity with paraparesis in combination with an afferent ataxia and bimalleolar reduced vibration sensation. The dysarthria was classified as pseudobulbar. Muscular reflexes were brisk in upper and lower limbs with a positive Babinski sign bilaterally. SPRS (Spastic Paraplegia Rating Scale) score was 19/52 points. The family history was negative for any neurological disorder.

Family 17, individual II.4 [c.655+1G>A, p.?]

This 67-year-old male of German descent (no evidence of consanguinity) presented with a 20-year history of deteriorating gait. His family history was positive for gait disorders. His mother was affected by a spastic gait disorder. Two of his brothers also showed a gait disorder, which was attributed to spasticity in the older and to peripheral neuropathy in the younger. Two maternal aunts had walking difficulties. One was classified as gait disorder due to peripheral neuropathy. The other one was classified as spastic gait disorder. Her son also had a spastic gait disorder.

The index patient reported normal cognitive development, but possibly delayed motor development with impaired sportive skills compared to his peers. Clear deterioration of gait was mentioned from the age of 43 years with recurrent falls. From the age of 60 years, he noticed dysarthria, followed by deterioration of fine motor skills. He suffered from hypacusis since the age of 30. He also developed urinary urge incontinence and intermitted faecal urge incontinence. At the age of 67, he was still able to walk freely for 10 meters. He required antispastic and anticholinergic therapy.

Neurological examination at the age of 67 years revealed mild dysarthria (unclassified) combined with a pyramidal syndrome, comprising of lower limb predominant spastic tetraparesis and unsustained ankle clonus. He showed contractures of the hips, knee extensors and bilateral pes equinus. The short extensor muscles of the toes were atrophic. The stretch reflexes of the upper limbs were normal. At the lower limbs, the patellar tendon reflex was brisk, while the Achilles tendon reflex was normal. The Trömner sign was positive at the left hand, the Babinski sign was negative. In addition, there was distal symmetric reduced surface sensation with painful dysesthesia of the limbs. Vibration sense was decreased at the lower limbs, but joint position sense was normal. Additionally, the patient showed gait and stance ataxia as well as postural instability. Furthermore, the patient had scoliosis.

Neurophysiological examinations confirmed sensory polyneuropathy and upper motor neuron affection, indicated by prolonged central motor conduction latency to the left leg.

Family 18, individual I.2 [c.655+1G>A, p.?]

This 77-year-old female of German descent presented with a 10-year history of progressive imbalance with repeated falls. Her family history was positive for gait disorders. Two of her sons developed walking difficulties in the mid-forties, with one of them showing spastic tetraparesis.

She had normal motor development despite alleged clumsiness since childhood. The first symptoms were disturbed balance and repeated falls at the age of 67 years. Because of her repeated falls, she was using a walker for a few months. At the age of 63, she developed urinary urge incontinence. As comorbidity, she reported arterial hypertension and bilateral glaucoma since the age of 72. One of her sons also suffered from glaucoma.

Examination revealed decreased vibration sense and ataxia of gait and stance, compatible with afferent ataxia. She had brisk stretch reflexes of the upper limbs, but no further indication for pyramidal affection. In contrast, the stretch reflexes of the lower limbs were reduced. She reported having problems to swallow since the age of 72 but clinically no dysphagia or dysarthria was detected. No additional examinations were performed.

Family 18, individual II.2 [c.655+1G>A, p.?]

This 60-year-old male of German descent presented with a 16-year history of slowly deteriorating gait. His family history was positive for gait disorders, without evidence of consanguinity. His mother suffered from repeated falls attributed to severe afferent ataxia and his younger brother also had a gait disorder.

He had a normal motor development and no cognitive impairment. His first symptoms at the age of 44 years consisted in the progressive feeling of stiffness and impaired coordination of the left leg. At the age of 60 years,

he was able to walk freely for 500 meters without pause. He was not using antispastic medication. He needed to use walking sticks only for longer walking distances. He developed a combined urinary dysfunction with both urinary urgency and impaired urinary voiding. As comorbidity, he reported long standing bilateral glaucoma. His mother also suffered from glaucoma.
Neurological examination revealed a pyramidal syndrome with lower limb predominant spastic tetraparesis, unsustained ankle clonus and bilateral positive Babinski sign. His gait was spastic-ataxic, with decreased vibration sense and impaired joint position sense, but absent cerebellar signs suggesting afferent ataxia.
Neurophysiology revealed sensory neuropathy. The motor evoked potentials showed a normal central motor conduction latency, while the sensory evoked potentials showed a prolonged latency to both upper limbs and the right leg and were absent to the left leg. MRI of the brain and the cervical spine at the age of 52 showed no significant pathology or atrophy. Routine examination of the CSF revealed no pathologies.

Family 18, individual II.3 [c.655+1G>A, p.?]

This 55-year-old male of German descent presented with a 15-year history of walking difficulty. His family history was positive for gait disorders, with his mother suffering from repeated falls from the age of 67 years and his older brother developing deteriorating gait from the age of 44 years.
He had a normal motor development and no cognitive impairment. His first symptoms at the age of 40 years consisted in stumbling and the progressive feeling of stiffness and impaired coordination of the left leg. At the age of 50 years, he was unlimited in his walking distance and still able to run. He was not using antispastic medication, but magnesium because of repeated cramps in the lower limbs. He reported normal bladder function.
Examination revealed brisk stretch reflexes of the lower limbs and unsustained ankle clonus at the right side, but no additional signs of a pyramidal syndrome. The left Achilles tendon reflex was reduced and the left S1 dermatome was hypaesthetic, both attributable to lumbar disc herniation. Vibration sense was decreased at both ankles.
Neurophysiology showed normal motor evoked potentials to the upper and lower limbs and normal nerve conductions studies.

Family 19, individual III.5 [c.655+1G>A, p.?]

This 70-year-old male of Russian and Baltic descent presented with a 25-year history of walking disturbances and repeated falls. His family history was positive for gait disorders. His mother and maternal grandmother as well as his two brothers and two sisters developed severe gait problems at the age of 50 to 60 years, with one of his brothers eventually becoming wheelchair bound.
He reported a normal motor development and no cognitive complaints. His first symptoms included progressive imbalance and stiffness of the legs. He worked as a construction worker, retiring early at the age of 62 due to balance problems. At the age of 65 years, he first noticed urinary urgency. He developed dysarthria without dysphagia and a disabling tremor of the upper limbs, which impaired eating, dressing, and writing at age of 67 years. At age of 70 years, he was still able to walk freely for 100 metres, with walking sticks the maximum walking distance was 400 metres, and he could stand unaided.
Neurological examination at age 70 revealed a pyramidal syndrome with lower limb predominant tetraspasticity with normal stretch reflexes of the upper and brisk reflexes to the lower limbs. Additionally, there was a cerebellar syndrome with moderate cerebellar dysarthria combined with saccadic pursuit and hypometric saccades, severe limb ataxia of the upper limbs with postural and kinetic tremor, mild ataxia of the lower limbs and moderate ataxia of stance. The gait was predominantly spastic. There was a distal symmetric sensory deficit, with severely decreased vibration sense of the lower limbs. In addition, there was bilateral ptosis.
Neurophysiological examination confirmed mixed sensory and motor neuropathy. The evoked motor potentials were prolonged to the upper limbs and absent to the lower limbs. The sensory evoked potentials (of the lower limbs) were absent. MRI of the brain and cervical spine showed moderate subcortical atherosclerotic encephalopathy at the age of 66 years, without significant atrophy of the cerebellum or the cervical spinal cord. Routine CSF parameters were normal.

Family 20, individual II.2 und II.3 [c.655+3A>C, p.?]

This family consists of two similarly affected brothers. The 58-year-old index patient first noticed a disturbed gait at the age of 40. His gait worsened slightly in the following years and his speech became slurred.
Neurological examination revealed spasticity of the lower extremities, mild unilateral ptosis and nystagmus, dysarthric speech, as well as mild cerebellar symptoms. Muscle reflexes were brisk. The patient walked with a spastic-atactic gait. The course of the disease was slowly progressive.
Family history was initially reported negative. However, examination revealed similar, but milder clinical findings in the index patient's older brother.

5. Consortia and associated partners

Solve-RD consortium

EKUT: Olaf Riess[1, 2], Tobias B. Haack[1, 2], Holm Graessner[1, 2], Stephan Ossowski[1, 3, 4], Birte Zurek[1, 2], Kornelia Ellwanger[1, 2], German Demidov[1], Marc Sturm[1], Joohyun Park[1], Leon Schütz[1], Julia M. Schulze-Hentrich[1, 5], Rebecca Schüle[6, 7], Jishu Xu[6, 8], Melanie Kellner[6, 8], Baptist Resch[7], Ingrid Kolen[7], Matthis Synofzik[6, 8], Carlo Wilke[6, 8], Andreas Traschütz[6, 8], Danique Beijer[9], Peter Heutink[6, 8], Ludger Schöls[6, 8], Holger Hengel[6, 8], Holger Lerche[10], Christian Boßelmann[10], Josua Kegele[10], Robert Lauerer-Braun[10], Stephan Lauxmann[10]

RUMC: Han Brunner[11-13], Hans Scheffer[11, 12], Nicoline Hoogerbrugge[11, 14], Alexander Hoischen[11, 14, 15], Peter A.C. 't Hoen [14, 16], Lisenka E.L.M. Vissers[11, 12], Christian Gilissen[11, 14], Wouter Steyaert[11, 14], Karolis Sablauskas[11], Richarda M. de Voer[11, 14], Erik-Jan Kamsteeg[11], Bart van de Warrenburg[12, 17], Nienke van Os[12, 17], Iris te Paske[11, 14], Erik Janssen[11, 14], Elke de Boer[11, 12], Marloes Steehouwer[11], Burcu Yaldiz[11], Kornelia Neveling[11], Bart van der Sanden[11], Lydia Sagath[11], Tjitske Kleefstra[11, 12]

University of Leicester: Anthony J. Brookes[18], Spencer Gibson[18], Umar Riaz[18], Greg Warren[18], Sai Anuhya Nalagandla[18], Yunze Patrick Wang[18], Deepthi Sukumaran[18], Sadegh Abadijou[18]

UNEW: Ana Töpf[19], Volker Straub[19], Chiara Marini Bettolo[19], Jordi Diaz Manera[19], Sophie Hambleton[20], Karin Engelhardt[20]

MUH: Jill Clayton-Smith[21, 22], Siddharth Banka[21, 22], Elizabeth Alexander[22], Adam Jackson[21, 22]

DIJON: Laurence Faivre[23-27], Christel Thauvin[23-27], Antonio Vitobello[25], Anne-Sophie Denommé-Pichon[25], Yannis Duffourd[25, 26], Ange-Line Bruel[25], Victor Couturier[25]

CNAG-CRG: Sergi Beltran[28, 29], Ivo Glynne Gut[28, 30], Steven Laurie[28, 30], Davide Piscia[28], Leslie Matalonga[28, 30], Anastasios Papakonstantinou[28, 30], Gemma Bullich[28, 30], Alberto Corvo[28, 30], Marcos Fernandez-Callejo[28, 30], Carles Hernández[28, 30], Daniel Picó[28, 30], Ida Paramonov[28, 30], Anna Esteve Codina[28, 31], Marc Dabad[28], Marta Gut[28, 31], Emanuele Raineri[28], Hanns Lochmüller[28, 30]

EURORDIS: Gulcin Gumus[32], Virginie Bros-Facer[33]

INSERM-Orphanet: Ana Rath[34], Marc Hanauer[34], David Lagorce[34],Oscar Hongnat[34], Maroua Chahdil[34], Caterina Lucano[34], Emeline Lebreton[34]

INSERM-ICM: Giovanni Stevanin[35, 36], Alexandra Durr[35, 37], Claire-Sophie Davoine[35], Léna Guillot-Noel[35], Anna Heinzmann[35, 37], Giulia Coarelli[35, 37]

INSERM-CRM: Gisèle Bonne[38], Teresinha Evangelista[38, 39], Valérie Allamand[38], Isabelle Nelson[38**Fehler! Textmarke nicht definiert.**], Rabah Ben Yaou[38-40**Fehler! Textmarke nicht definiert.**], Corinne Metay[38, 41], Bruno Eymard[38, 39], Enzo Cohen[38], Antonio Atalaia[38], Tanya Stojkovic[38, 39]

Univerzita Karlova: Milan Macek Jr.[42], Marek Turnovec[42], Dana Thomasová[42], Radka Pourová Kremliková[42], Vera Franková[42], Markéta Havlovicová[42], Lukáš Ryba[42], Petra Lišková[43, 44], Pavla Doležalová[45], Alice Krebsová[46]

EMBL-EBI: Helen Parkinson[47], Thomas Keane[47], Mallory Freeberg[47], Coline Thomas[47], Dylan Spalding[47,48]

Jackson Laboratory: Peter Robinson[49, 50], Daniel Danis[49]

KCL: Glenn Robert[51], Alessia Costa[52]

UCL-IoN: Mike Hanna[53], Henry Houlden[54], Mary Reilly[53], Jana Vandrovcova[54], Stephanie Efthymiou[54], Heba Morsy[54, 55], Elisa Cali[54], Francesca Magrinelli[56], Sanjay M. Sisodiya[57], Ravishankara Bellampalli[57], Patrick Moloney[57], Jonathan Rohrer[58]

UCL-ICH, Francesco Muntoni[59, 60], Irina Zaharieva[59], Anna Sarkozy[59], Luke Perry[59, 60], Veronica Pini[59], Juliane Müller[59]

Universiteit Antwerpen: Vincent Timmerman[61, 62], Jonathan Baets[63, 64], Geert de Vries[62, 63], Jonathan De Winter[62-64], Peter de Jonghe[62, 64], Liedewei Van de Vondel[61-63], Willem De Ridder[62-64], Sarah Weckhuysen[63-65], Hannah Stamberger[64, 65], Charissa Millevert[64, 65], Noor Smal[65]

Uni Naples/Telethon UDP: Vincenzo Nigro[66, 67], Manuela Morleo[66, 67], Michele Pinelli[67], Sandro Banfi[66, 67], Annalaura Torella[66, 67], Roberta Zeuli[66], Mariateresa Zanobio[66], Giulio Piluso[66]

UNIFE: Alessandra Ferlini[68], Rita Selvatici[68], Francesca Gualandi[68], Stefania Bigoni[68], Marcella Neri[68]

UKB: Stefan Aretz[69, 70], Isabel Spier[69, 70], Anna Katharina Sommer[69], Sophia Peters[69]

IPATIMUP: Carla Oliveira[71-73], Jose Garcia-Pelaez[71, 72], Rita Barbosa-Matos[71, 72], Celina São José[71, 72] , Marta Ferreira[71, 72], Irene Gullo[71-74], Susana Fernandes[75], Luzia Garrido[74], Pedro Ferreira[71, 72, 76], Fátima Carneiro[71-74]

UMCG: Morris A Swertz[77], Lennart Johansson[77], Joeri K van der Velde[77], Gerben van der Vries[77], Pieter B Neerincx[77], Dieuwke Roelofs-Prins[77], David Ruvolo[77], Marielle van Gijn[78, 79]

Kristin M Abbott[78], Wilhemina S Kerstjens Frederikse[78], Eveline Zonneveld-Huijssoon[78]

Charité: Sebastian Köhler[80]

SHU: Alison Metcalfe[51, 81], Richard Moore[82]

APHP: Alain Verloes[83, 84], Séverine Drunat[83, 84], Delphine Heron[85, 86], Cyril Mignot[85, 87], Boris Keren[85], Jean-Madeleine de Sainte Agathe[85]

Associated partners

Rami Abou Jamra[88], Marc Abramowicz[89, 90], Özge Aksel Kiliçarslan[91], Nicholas Allen[92], Francisco Javier Alonso García de la Rosa[93], Simona Balestrini[94], Peter Balicza[95], Tobias Bartolomaeus[88], Ayşe Nazlı Başak[96], Laura Batlle Masó[97, 98], David Beeson[99], Valerie Benoit[100], Katherine Benson[101], Eva Bermejo Sánchez[93], Emilia K. Bijlsma[102], Elke Bogaert[103], Mara Bourbouli[104], Kaan Boztug[105-109], Sylvain Brohée[100], Susan Byrne[110-112], Andrés Caballero Garcia de Oteyza[113, 114], Gabriel Capella[115, 116], Evelina Carpancea[117], Gianpiero Cavalleri[101, 110, 118], Ana Cazurro-Gutiérrez[119], Patrick F. Chinnery[120, 121], Maria-Roberta Cilio[117], Andrea Ciolfi[122], Kristl Claeys[123, 124], Roger Colobran[125-127], Isabell Cordts[128], Judith Cossins[99], Karin Dahan[100, 129], Bruno Dallapiccola[122], Norman Delanty[101, 110, 130], Christel Depienne[131, 132], Chantal Depondt[133], Bart Dermaut[103, 134, 135], Marcus Deschauer[128], Julie Desir[100], Anne Destrée[100], Minas Drakos[104], Sarah Duerinckx[133], Berta Estevez[136, 137], Athanasios Evangeliou[138], Chiara Fallerini[139, 140], Marco Ferilli[122], Simone Furini[139, 140], Julien Gagneur[141-143], Hamidah Ghani[101, 110, 118], Marie Greally[101, 110, 144], Bodo Grimbacher[114, 145-148], Renzo Guerrini[149], Peter Hackman[150], Matthias Haimel[105-107], Eva Hammar Bouveret[89], Dimitri Hemelsoet[134, 151], Rebecca Herzog[152, 153], Mariette J.V. Hoffer[102], Elke Holinski-Feder[154], Rita Horvath[120], Manon Huibers[155], Michele Iacomino[156, 157], Mridul Johari[150], Elisabeth Kapaki[158], Deniz Karadurmus[100], Mert Karakaya[159-162], Evgenia Kokosali[104], Christian Korff[163], Leon Krass[141-143], Didier Lacombe[164], Andreas Laner[165], Helen Leavis[166], Damien Lederer[167], Elsa Leitão[131], Hanns Lochmüller[91, 168, 169], Katja Lohmann[170], Estrella López Martín[93], Rebeka Luknárová[141], Alfons Macaya[119, 171], Sivasankar Malaichamy[91], Anna Marcé-Grau[119], Beatriz Martínez Delgado[93], Sandrine Mary[167], Frédéric Masclaux[89], Lambros Mathioudakis[104], Ales Maver[172], Patrick May[173], Isabelle Maystadt[100, 174], Davide Mei[94], Christian Mertes[141, 142], Colombine Meunier[100], Maria Judit Molnar[95], Olivier Monestier[100], Stéphanie Moortgat[100], Alexander Münchau[152, 175], Francina Munell[119], Andrés Nascimento Osorio[136, 176, 177], Daniel Natera de Benito[136, 176, 177], Mary O Reghan[112], Catarina Olimpio[120, 178], Elena Parrini[94], Martje Pauly[152, 170], Belén Pérez-Dueñas[119], Borut Peterlin[172], Konrad Platzer[88], Kiran Polavarapu[91], Bruce Poppe[103, 134, 135], Manuel Posada De la Paz[93], Flavia Privitera[139, 140], Francesca Clementina Radio[122], Thiloka Ratnaike[179], Alessandra Renieri[139, 140, 180], Antonella Riva[156, 181], Caroline Rooryck[164], Andreas Roos[91, 182], Claudia A.L. Ruivenkamp[102], Andreas Rump[183, 184], Gijs W.E. Santen[102], Marco Savarese[150], Marcello Scala[181, 185], Katherine Schon[120, 178], Evelin Schröck[183], Nika Schuermans[103, 134, 135], Paolo Scudieri[156, 181], Martha Spilioti[186], Verena Steinke-Lange[154], Pasquale Striano[181, 187], Yves Sznajer[188], Marco Tartaglia[122], Rachel Thompson[91], Aurelien Trimouille[189], Bjarne Udd[150, 190, 191], Paolo Uva[157], Laura Valle[115, 116], Lars van der Veken[155], Roxane van Heurck[89], Joris van Montfrans[192], Erika Van Nieuwenhove[192], Hannah Verdin[103], David Webb[112], Brunhilde Wirth[159-162], Vicente A. Yépez[141], Ioannis Zaganas[104], Federico Zara[156, 181], Kristina Zguro[139, 140].

Affiliations

1. Institute of Medical Genetics and Applied Genomics, University of Tübingen, Tübingen, Germany.
2. Centre for Rare Diseases, University of Tübingen, Tübingen, Germany.
3. NGS Competence Center Tübingen (NCCT), University of Tübingen, Tübingen, Germany.
4. Institute for Bioinformatics and Medical Informatics (IBMI), University of Tübingen, Tübingen, Germany.
5. Department of Genetics/Epigenetics, Faculty NT, Saarland University, Saarbrücken, Germany.
6. Department of Neurodegeneration, Hertie Institute for Clinical Brain Research (HIH), University of Tübingen, Tübingen, Germany.
7. Division of Neurodegenerative Diseases and Movement Disorders, Department of Neurology, University of Heidelberg, Heidelberg, Germany.
8. German Center for Neurodegenerative Diseases (DZNE), Tübingen, Germany.
9. Division Translational Genomics of Neurodegenerative Diseases, Hertie-Institute for Clinical Brain Research and Center of Neurology, University of Tübingen, Tübingen, Germany.
10. Department of Neurology and Epileptology, Hertie Institute for Clinical Brain Research (HIH), University of Tübingen, Tübingen, Germany.
11. Department of Human Genetics, Radboud University Medical Center, Nijmegen, The Netherlands.
12. Donders Institute for Brain, Cognition and Behaviour, Radboud University Medical Center, Nijmegen, The Netherlands.
13. Department of Clinical Genetics, Maastricht University Medical Centre, Maastricht, the Netherlands.
14. Radboud Institute for Molecular Life Sciences, Nijmegen, The Netherlands.
15. Department of Internal Medicine and Radboud Center for Infectious Diseases (RCI), Radboud University Medical Center, Nijmegen, the Netherlands.

16. Center for Molecular and Biomolecular Informatics, Radboud University Medical Center, Nijmegen, the Netherlands.
17. Department of Neurology, Radboud University Medical Center, Nijmegen, The Netherlands.
18. Department of Genetics and Genome Biology, University of Leicester, Leicester, UK.
19. John Walton Muscular Dystrophy Research Centre, Translational and Clinical Research Institute, Newcastle University and Newcastle Hospitals NHS Foundation Trust, Newcastle upon Tyne, UK.
20. Primary Immunodeficiency Group, Translational and Clinical Research Institute, Newcastle University and Newcastle upon Tyne Hospitals NHS Foundation Trust, Newcastle upon Tyne, UK.
21. Division of Evolution, Infection and Genomics, School of Biological Sciences, Faculty of Biology, Medicine and Health, University of Manchester, Manchester M13 9WL, UK.
22. Manchester Centre for Genomic Medicine, St Mary's Hospital, Manchester University Hospitals NHS Foundation Trust, Health Innovation Manchester, Manchester M13 9WL, UK.
23. Dijon University Hospital, Genetics Department, Dijon, France.
24. Dijon University Hospital, Centre of Reference for Rare Diseases: Development disorders and malformation syndromes, Dijon, France.
25. Inserm - University of Burgundy-Franche Comté, UMR1231 GAD, Dijon, France.
26. Dijon University Hospital, FHU-TRANSLAD, Dijon, France.
27. Dijon University Hospital, GIMI institute, Dijon, France.
28. Centro Nacional de Análisis Genómico (CNAG), C/Baldiri Reixac 4, 08028 Barcelona, Spain.
29. Departament de Genètica, Microbiologia i Estadística, Facultat de Biologia, Universitat de Barcelona (UB), Barcelona, Spain.
30. Universitat de Barcelona (UB), Barcelona, Spain.
31. Universitat Pompeu Fabra (UPF), Barcelona, Spain.
32. EURORDIS-Rare Diseases Europe, Sant Antoni Maria Claret 167 - 08025 Barcelona, Spain.
33. EURORDIS-Rare Diseases Europe, Plateforme Maladies Rares, 75014 Paris, France.
34. INSERM, US14 - Orphanet, Plateforme Maladies Rares, 75014 Paris, France.
35. Institut du Cerveau, INSERM U1127, CNRS UMR7225, Sorbonne university, Paris, France
36. INCIA, EPHE, CNRS UMR5287, Bordeaux university, Bordeaux, France.
37. Hôpital de la Pitié-Salpêtrière, Assistance Publique-Hôpitaux de Paris (AP-HP), Paris, France.
38. Sorbonne Université, Inserm, Institut de Myologie, Centre de Recherche en Myologie, F-75013 Paris, France.
39. AP-HP, Centre de Référence de Pathologie Neuromusculaire Nord, Est, Ile-de-France, Institut de Myologie, G.H. Pitié-Salpêtrière, F-75013 Paris, France.
40. Institut de Myologie, Equipe Bases de données, G.H. Pitié-Salpêtrière, F-75013 Paris, France.
41. AP-HP, Unité Fonctionnelle de Cardiogénétique et Myogénétique Moléculaire et Cellulaire, G.H. Pitié-Salpêtrière, F-75013 Paris, France.
42. Department of Biology and Medical Genetics, Charles University Prague-2nd Faculty of Medicine and University Hospital Motol, Prague, Czech Republic.
43. Department of Paediatrics and Inherited Metabolic Disorders, First Faculty of Medicine, Charles University and General University Hospital in Prague, Prague, Czech Republic.
44. Department of Ophthalmology, First Faculty of Medicine, Charles University and General University Hospital in Prague, Prague, Czech Republic.
45. Centre for Paediatric Rheumatology and Autoinflammatory Diseases, Department of Paediatrics and Inherited Metabolic Disorders, 1st Faculty of Medicine, Charles University and General University Hospital in Prague, Czech Republic.
46. Department of Cardiology - Institute of Clinical and Experimental Medicine and Department of Biology and Medical Genetics - 2nd Faculty of Medicine Charles University, Prague, Czech Republic
47. European Bioinformatics Institute, European Molecular Biology Laboratory, Wellcome Genome Campus, Hinxton, Cambridge, United Kingdom.
48. CSC–IT Center for Science, 02101 Espoo, Finland.
49. Jackson Laboratory for Genomic Medicine, Farmington, CT 06032, USA.
50. Berlin Institute of Health at Charité – Universitätsmedizin Berlin, Charitéplatz 1, 10117 Berlin, Germany
51. Florence Nightingale Faculty of Nursing, Midwifery & Palliative Care, King's College, London, UK.
52. Society and Ethics Research, Connecting Science, Wellcome Genome Campus, Hinxton, UK.
53. MRC Centre for Neuromuscular Diseases and National Hospital for Neurology and Neurosurgery, UCL Queen Square Institute of Neurology, London, UK.
54. Department of Neuromuscular Diseases, UCL Queen Square Institute of Neurology, London, UK.
55. Department of Human Genetics, Medical Research Institute, Alexandria University, Egypt.

56. Department of Clinical and Movement Neurosciences, UCL Queen Square Institute of Neurology, University College London, WC1N 3BG.
57. Department of Clinical and Experimental Epilepsy, UCL Queen Square Institute of Neurology, London, UK.
58. Dementia Research Centre, Department of Neurodegenerative Disease, UCL Queen Square Institute of Neurology, London, UK.
59. Dubowitz Neuromuscular Centre, UCL Great Ormond Street Hospital, London, UK.
60. NIHR Great Ormond Street Hospital Biomedical Research Centre, London, United Kingdom.
61. Peripheral Neuropathy Research Group, University of Antwerp, Antwerp, Belgium.
62. Laboratory of Neuromuscular Pathology, Institute Born-Bunge, University of Antwerp, Antwerpen, Belgium.
63. Translational Neurosciences, Faculty of Medicine and Health Sciences, University of Antwerp, Belgium.
64. Neuromuscular Reference Centre, Department of Neurology, Antwerp University Hospital, Antwerpen, Belgium.
65. VIB-CMN, Applied and Translational Neurogenomics Group.
66. Dipartimento di Medicina di Precisione, Università degli Studi della Campania "Luigi Vanvitelli", Napoli, Italy.
67. Telethon Institute of Genetics and Medicine, Pozzuoli, Italy.
68. Unit of Medical Genetics, Department of Medical Sciences, University of Ferrara, Italy.
69. Institute of Human Genetics, Medical Faculty, University of Bonn, Bonn, Germany.
70. Center for Hereditary Tumor Syndromes, University Hospital Bonn, Bonn, Germany.
71. i3S - Instituto de Investigação e Inovação em Saúde, Universidade do Porto, Portugal.
72. IPATIMUP - Institute of Molecular Pathology and Immunology of the University of Porto, Portugal.
73. Faculty of Medicine, University of Porto, Portugal.
74. CHUSJ, Centro Hospitalar e Universitário de São João, Porto, Portugal.
75. Departament of Genetics, Faculty of Medicine, University of Porto, Portugal.
76. Faculty of Sciences, University of Porto, Portugal.
77. Department of Genetics, Genomics Coordination Center, University Medical Center Groningen, University of Groningen, Groningen, The Netherlands.
78. Department of Genetics, University Medical Center Groningen, University of Groningen, Groningen, The Netherlands.
79. Department of Human Genetics, Amsterdam UMC, University of Amsterdam, the Netherlands.
80. Ada Health GmbH, Karl-Liebknecht-Str. 1, 10178 Berlin, Germany.
81. College of Health, Well-being and Life-Sciences, Sheffield Hallam University, Sheffield, UK.
82. Advanced Wellbeing Research Centre, Sheffield Hallam University, Olympic Legacy Park, 2 Old Hall Road, Sheffield, S9 3TU.
83. Dept of Genetics, Assistance Publique-Hôpitaux de Paris - Université de Paris, Robert DEBRE University Hospital, 48 bd SERURIER, Paris, France.
84. INSERM UMR 1141 "NeuroDiderot", Hôpital Robert DEBRE, Paris, France.
85. Department of Genetics, Assistance Publique-Hôpitaux de Paris - Sorbonne Université, Pitié-Salpêtrière University Hospital, 83 Boulevard de l'Hôpital, Paris, France.
86. Reference center of rare diseases "intellectuel disability of rare causes", Paris, France.
87. Institut du Cerveau (ICM), UMR S 1127, Inserm U1127, CNRS UMR 7225, Sorbonne Université, 75013, Paris, France.
88. Institute of Human Genetics, University of Leipzig Medical Center, Leipzig, Germany.
89. Genetic Medicine Division, University Hospitals and University of Geneva, Geneva, Switzerland.
90. Genetics & Development, Faculty of Medicine, University of Geneva, Geneva, Switzerland.
91. Children's Hospital of Eastern Ontario Research Institute, University of Ottawa, Ottawa, Canada.
92. Paediatric Neurology, University Hospital Galway.
93. Institute of Rare Diseases Research, Spanish Undiagnosed Rare Diseases Cases Program (SpainUDP) & Undiagnosed Diseases Network International (UDNI), Instituto de Salud Carlos III, Madrid, Spain.
94. Neuroscience Department, Children's Hospital A. Meyer-University of Florence, 50139, Florence, Italy.
95. Institute of Genomic Medicine and Rare Diseases, Semmelweis University, Budapest, Hungary.
96. Koç Universıty,School of Medicine, Translational Medicine Research Center, KUTTAM-NDAL Istanbul Turkey.
97. Infection in Immunocompromised Pediatric Patients Research Group, Vall d'Hebron Research Institute (VHIR), Barcelona, Spain.
98. Pediatric Infectious Diseases and Immunodeficiencies Unit, Vall d'Hebron University Hospital (HUVH),Barcelona, Spain.

99. Nuffield Department of Clinical Neurosciences, University of Oxford, UK.
100. Centre de Génétique Humaine, Institut de Pathologie et de Génétique, Gosselies, Belgium.
101. School of Pharmacy and Bimolecular Sciences, RCSI, Dublin, Ireland.
102. Department of Clinical Genetics, Leiden University Medical Center, Leiden, The Netherlands.
103. Center for Medical Genetics, Ghent University Hospital, Ghent, Belgium.
104. Neurology / Neurogenetics Laboratory University of Crete, Heraklion, Crete, Greece.
105. Ludwig Boltzmann Institute for Rare and Undiagnosed Diseases, Vienna, Austria.
106. St. Anna Children's Cancer Research Institute (CCRI), Vienna, Austria.
107. CeMM Research Center for Molecular Medicine of the Austrian Academy of Sciences, Vienna, Austria.
108. Department of Pediatrics and Adolescent Medicine, Medical University of Vienna, Vienna, Austria.
109. St. Anna Children's Hospital, Department of Pediatrics and Adolescent Medicine, Medical University of Vienna, Vienna, Austria.
110. SFI FutureNeuro Research Centre, Dublin, Ireland.
111. Department of Paediatrics, RCSI, Ireland.
112. Department of Paediatrics Neurology, CHI, Dublin,Ireland.
113. Institute for Immunodeficiency, Center for Chronic Immunodeficiency (CCI), Medical Center, Faculty of Medicine, Albert-Ludwigs-University of Freiburg, Germany.
114. RESIST – Cluster of Excellence 2155 to Hanover Medical School, Satellite Center Freiburg, Germany.
115. Bellvitge Biomedical Research Institute (IDIBELL), Barcelona, Spain.
116. Catalan Institute of Oncology (IROCA), Barcelona, Spain.
117. Pediatric Neurology Department, Saint-Luc University Hospital, Université Catholique de Louvain, Brussels, Belgium.
118. SFI Centre for Research Training in Genomics Data Science, Ireland.
119. Pediatric Neurology Research Group, Vall d'Hebron Research Institute, Universitat Autònoma de Barcelona, Barcelona, Spain.
120. Department of Clinical Neurosciences, University of Cambridge, Cambridge, UK.
121. Medical Research Council Mitochondrial Biology Unit, University of Cambridge, Cambridge, UK.
122. Molecular Genetics and Functional Genomics, Ospedale Pediatrico Bambino Gesù, IRCCS, Rome, Italy.
123. Department of Neurology, University Hospitals Leuven, Leuven, Belgium.
124. Laboratory for Muscle Diseases and Neuropathies, Department of Neurosciences, and Leuven Brain Institute (LBI), KU Leuven - University of Leuven, Leuven, Belgium.
125. Diagnostic Immunology Research Group, Vall d'Hebron Research Institute (VHIR), Barcelona, Spain.
126. Immunology Division, Genetics Department. Vall d'Hebron University Hospital (HUVH), Barcelona, Spain.
127. Immunology Unit. Department of Cell Biology, Physiology and Immunology. Autonomous University of Barcelona (UAB), Bellaterra, Spain.
128. Department of Neurology, Klinikum rechts der Isar, Technical University Munich, Munich, Germany.
129. Département de néphrologie, Cliniques Universitaires Saint-Luc, Bruxelles, Belgium.
130. Department of Neurology, Beaumont Hospital, Dublin, Ireland.
131. Institute of Human Genetics, University Hospital Essen, University Duisburg-Essen, Essen, Germany.
132. Institut du Cerveau et de la Moelle épinière (ICM), Sorbonne Université, UMR S 1127, Inserm U1127, CNRS UMR 7225, F-75013 Paris, France.
133. Department of Neurology, CUB Erasme Hospital, Hôpital Universitaire de Bruxelles, Université Libre de Bruxelles, Brussels, Belgium.
134. Program for Undiagnosed Rare Diseases (UD-PrOZA), Ghent University Hospital, Ghent, Belgium.
135. Department of Biomolecular Medicine, Faculty of Medicine and Health Sciences, Ghent University, Ghent, Belgium.
136. Neuromuscular Disorders Unit , Department of Pediatric Neurology. Hospital Sant Joan de Déu, Barcelona, Spain
137. Laboratory of Neurogenetics and Molecular Medicine - IPER, Institut de Recerca Sant Joan de Déu, Barcelona, Spain.
138. Saint Luke Hospital, Division of Child Neurology, Thessaloniki, Greece.
139. Med Biotech Hub and Competence Center, Department of Medical Biotechnologies, University of Siena, Italy.
140. Medical Genetics, University of Siena, Italy.
141. School of Computation, Information and Technology, Technical University of Munich, Garching, Germany.
142. Institute of Human Genetics, School of Medicine, Technical University of Munich, Munich, Germany.
143. Computational Health Center, Helmholtz Center Munich, Neuherberg, Germany.
144. Department of Clinical Genetics, CHI, Dublin, Ireland.

145. Institute for Immunodeficiency, Center for Chronic Immunodeficiency (CCI), Medical Center, Faculty of Medicine, Albert-Ludwigs-University of Freiburg, Germany.
146. Clinic of Rheumatology and Clinical Immunology, Center for Chronic Immunodeficiency (CCI), Medical Center, Faculty of Medicine, Albert-Ludwigs-University of Freiburg, Germany.
147. DZIF – German Center for Infection Research, Satellite Center Freiburg, Germany.
148. CIBSS – Centre for Integrative Biological Signalling Studies, Albert-Ludwigs University, Freiburg, Germany.
149. Neurofarba Department, University of Florence, Florence, Italy.
150. Folkhälsan Research Centre and Medicum, University of Helsinki, Helsinki, Finland.
151. Dpt. of Neurology, Ghent University Hospital, Ghent, Belgium.
152. Institute of Systems Motor Science, University of Lübeck, Ratzeburger Allee 160, 23562, Lübeck, Germany.
153. Department of Neurology, University Hospital Schleswig Holstein, Ratzeburger Allee 160, 23538, Lübeck, Germany.
154. Medizinische Klinik und Poliklinik IV – Campus Innenstadt, Klinikum der Universität München, Munich, Germany.
155. Department of Genetics, Division Laboratories, Pharmacy and Biomedical Genetics, University Medical Center Utrecht, Utrecht University, Utrecht, the Netherlands.
156. Unit of Medical Genetics, IRCCS Istituto Giannina Gaslini, Genoa, Italy.
157. Clinical Bioinformatics, IRCCS Istituto Giannina Gaslini, Genoa, Italy.
158. Neurochemistry and Biomarker Unit, 1st Department of Neurology, School of Medicine, National and Kapodistrian University of Athens, Eginition Hospital, Athens, Greece.
159. Institute of Human Genetics, University Hospital of Cologne, University Cologne, Kerpener Str. 34, 50931 Cologne, Germany.
160. Center for Molecular Medicine Cologne, University of Cologne, 50931 Cologne, Germany.
161. Institute for Genetics, University of Cologne, 50674 Cologne, Germany.
162. Center for Rare Diseases Cologne, University Hospital Cologne, 50937, Cologne, Germany.
163. Pediatric Neurology Unit, University Hospitals, Geneva, Switzerland.
164. Univ. Bordeaux, MRGM INSERM U1211, CHU de Bordeaux, Service de Génétique Médicale , F-33000 Bordeaux, France.
165. MGZ - Medical Genetics Center, Munich, Germany.
166. Department of Rheumatology & Clinical Immunology, University Medical Center Utrecht, Utrecht University, Utrecht, the Netherlands.
167. Institute of Pathology and Genetics, Charleroi, Belgium.
168. Department of Neuropediatrics and Muscle Disorders, Medical Center, Faculty of Medicine, University of Freiburg, Freiburg, Germany.
169. Centro Nacional de Análisis Genómico (CNAG-CRG), Center for Genomic Regulation, Barcelona Institute of Science and Technology (BIST), Barcelona, Spain.
170. Institute of Neurogenetics, University of Lübeck, Ratzeburger Allee 160, 23562, Lübeck, Germany.
171. Institut de Neurociències, Universitat Autònoma de Barcelona, Barcelona, Spain.
172. Clinical Institute of Genomic Medicine, University Medical Centre Ljubljana, Slovenia.
173. Luxembourg Centre for Systems Biomedicine, University of Luxembourg, Esch-sur-Alzette, Luxembourg.
174. Département de Médecine, Université de namur (Unamur), Namur, Belgique.
175. Center for Rare Diseases, University Hospital Schleswig-Holstein, Ratzeburger Allee 160, 23562, Lübeck, Germany.
176. Applied Research in Neuromuscular Diseases, Institut de Recerca Sant Joan de Déu, Barcelona, Spain.
177. Center for Biomedical Research Network on Rare Diseases (CIBERER), ISCIII.
178. East Anglian Medical Genetics Service, Cambridge University Hospitals NHS Foundation Trust, Cambridge, UK.
179. Department of Paediatrics, University of Cambridge, Cambridge, UK.
180. Genetica Medica, Azienda Ospedaliero-Universitaria Senese, Italy.
181. Department of Neurosciences, Rehabilitation, Ophthalmology, Genetics, Maternal and Child Health, University of Genoa, Genoa, Italy.
182. Department of Pediatric Neurology, Developmental Neurology and Social Pediatrics, Children's Hospital University of Essen, Essen, Germany.
183. Institute for Clinical Genetics, Faculty of Medicine Carl Gustav Carus, Technical University Dresden, Dresden, Germany.
184. Center for Personalized Oncology, University Hospital Carl Gustav Carus, Technical University Dresden, Dresden, Germany.
185. Pediatric Neurology and Muscular Disease Unit, IRCCS Istituto Giannina Gaslini, Genoa, Italy.

186. 1st Department of Neurology, Aristotle University of Thessaloniki, University General Hospital of Thessaloniki, AHEPA, Thessaloniki, Greece.
187. IRCCS Istituto Giannina Gaslini, Genoa, Italy.
188. Human Genetics Department, Saint-Luc University Hospital, Université Catholique de Louvain, Brussels, Belgium.
189. Laboratoire de Génétique Moléculaire, Service de Génétique Médicale, CHU Bordeaux – Hôpital Pellegrin, Place Amélie Raba Léon, 33076 Bordeaux Cedex, France.
190. Tampere Neuromuscular Center, Tampere, Finland.
191. Vasa Central Hospital, Vaasa, Finland.
192. Department of Pediatric Immunology and Infectious Diseases, University Medical Center Utrecht, Utrecht University, Utrecht, the Netherlands.
