# Supplementary material for: Loss-of-function variants in the CAPN1 activator CD99L2 cause X-linked spastic ataxia
Source: Nat Commun. 2026 Feb 14;17:1698. doi: 10.1038/s41467-026-69337-9 (PMC12910011; doi:10.1038/s41467-026-69337-9)
Supplement: Supplementary file 4 — Reporting Summary [file 41467_2026_69337_MOESM4_ESM.pdf]

nature portfolio

Corresponding author(s): *Tobias Haack, Jonasz Weber*

Last updated by author(s): *2025/11/23*

Reporting Summary

Nature Portfolio wishes to improve the reproducibility of the work that we publish. This form provides structure for consistency and transparency in reporting. For further information on Nature Portfolio policies, see our Editorial Policies and the Editorial Policy Checklist.

Statistics

For all statistical analyses, confirm that the following items are present in the figure legend, table legend, main text, or Methods section.

n/a	Confirmed	
☐	☒	The exact sample size (*n*) for each experimental group/condition, given as a discrete number and unit of measurement
☐	☒	A statement on whether measurements were taken from distinct samples or whether the same sample was measured repeatedly
☐	☒	The statistical test(s) used AND whether they are one- or two-sided<br>*Only common tests should be described solely by name; describe more complex techniques in the Methods section.*
☐	☒	A description of all covariates tested
☐	☒	A description of any assumptions or corrections, such as tests of normality and adjustment for multiple comparisons
☐	☒	A full description of the statistical parameters including central tendency (e.g. means) or other basic estimates (e.g. regression coefficient) AND variation (e.g. standard deviation) or associated estimates of uncertainty (e.g. confidence intervals)
☐	☒	For null hypothesis testing, the test statistic (e.g. *F*, *t*, *r*) with confidence intervals, effect sizes, degrees of freedom and *P* value noted<br>*Give P values as exact values whenever suitable.*
☒	☐	For Bayesian analysis, information on the choice of priors and Markov chain Monte Carlo settings
☒	☐	For hierarchical and complex designs, identification of the appropriate level for tests and full reporting of outcomes
☒	☐	Estimates of effect sizes (e.g. Cohen's *d*, Pearson's *r*), indicating how they were calculated

Our web collection on statistics for biologists contains articles on many of the points above.

Software and code

Policy information about availability of computer code

Data collection	No software was used for data collection
Data analysis	https://github.com/imgag/megSAP

For manuscripts utilizing custom algorithms or software that are central to the research but not yet described in published literature, software must be made available to editors and reviewers. We strongly encourage code deposition in a community repository (e.g. GitHub). See the Nature Portfolio guidelines for submitting code & software for further information.

Data

Policy information about availability of data

All manuscripts must include a data availability statement. This statement should provide the following information, where applicable:

- Accession codes, unique identifiers, or web links for publicly available datasets
- A description of any restrictions on data availability
- For clinical datasets or third party data, please ensure that the statement adheres to our policy

The majority of diagnostic-derived genetic datasets cannot be shared due to privacy concerns. Parts of the genome study cohort has been additionally consented for FAIR use of generated sequencing data. These data have been deposited at the German Human Genome Archive and are availably via controlled access:
1.)https://data.ghga.de/dataset/GHGAD30455110557096
2.)https://data.ghga.de/dataset/GHGAD63728375628676
From the generated RNA-seq data the read counts can be shared upon request.

Research involving human participants, their data, or biological material

Policy information about studies with human participants or human data. See also policy information about sex, gender (identity/presentation), and sexual orientation and race, ethnicity and racism.

Reporting on sex and gender	The terms sex/gender are appropriately used in the manuscript. No sex/gender-specific research has been performed.
Reporting on race, ethnicity, or other socially relevant groupings	not applicable
Population characteristics	Details on the study cohort are provided in the manuscript and supplementary material
Recruitment	These were consecutive individuals sent for diagnostic testing; part of the cohort has been included in the "Genome-first" and "Ge-Med" studies.
Ethics oversight	Ethics committee of the University of Tuebingen

Note that full information on the approval of the study protocol must also be provided in the manuscript.

Field-specific reporting

Please select the one below that is the best fit for your research. If you are not sure, read the appropriate sections before making your selection.

☒ Life sciences ☐ Behavioural & social sciences ☐ Ecological, evolutionary & environmental sciences

For a reference copy of the document with all sections, see nature.com/documents/nr-reporting-summary-flat.pdf

Life sciences study design

All studies must disclose on these points even when the disclosure is negative.

Sample size	Sample sizes were not determined a priori by statistical methods. For functional analyses, we selected sample sizes in accordance with established practice in the field, guided by prior studies and the known variability of the respective assays.
Data exclusions	No datasets were excluded from the analyses. For functional experiments, individual data points identified as statistical outliers were removed according to the procedures outlined in the Statistics and Reproducibility section.
Replication	Training of the LASSO model was performed in the exome cohort and replicated in the genome cohort. For functional analyses, the number of independent experimental replicates is indicated in the respective figure legends. All reported findings were successfully reproduced.
Randomization	Randomization was not performed, as experimental conditions and sample allocation did not require random assignment.
Blinding	Blinding was not undertaken, as the study design and experimental procedures did not involve conditions in which investigator knowledge could influence data acquisition or analysis.

Reporting for specific materials, systems and methods

We require information from authors about some types of materials, experimental systems and methods used in many studies. Here, indicate whether each material, system or method listed is relevant to your study. If you are not sure if a list item applies to your research, read the appropriate section before selecting a response.

Materials & experimental systems

n/a	Involved in the study	
☐	☒	Antibodies
☐	☒	Eukaryotic cell lines
☒	☐	Palaeontology and archaeology
☒	☐	Animals and other organisms
☐	☒	Clinical data
☒	☐	Dual use research of concern
☒	☐	Plants

Methods

n/a	Involved in the study	
☒	☐	ChIP-seq
☒	☐	Flow cytometry
☒	☐	MRI-based neuroimaging

Antibodies

Antibodies used	A comprehensive list of antibodies, their dilutions, suppliers, and additional identifiers is given in the Methods section and the [illegible]

Supplementary Table 2, Supplementary Information file.
The following primary antibodies were employed in this study:
β-actin(AC-15/A5441, Sigma-Aldrich), Ataxin-3 (1H9/MAB5360, Merck), Calnexin (C4731, Sigma-Aldrich); CAPN1 (10538-1-AP, Proteintech) CAPN1 (ab39170, Abcam), CAPN2 (1E1F10/66977-1-Ig, Proteintech), CAPN2 (ab39165, Abcam), CAPN10 (ab28220, Abcam), CSS1 (P1/MAB3083, Merck), CAST (#4146S, Cell Signaling), CD99L2 (C-terminal) (HPA061400, Sigma-Aldrich), CD99L2 (N-terminal) (HPA038783, Sigma-Aldrich), FLAG (F7425, Sigma-Aldrich), GAPDH (0411/sc-47724, Santa Cruz), GAPDH (10494-1-AP, Proteintech), LC3B (#2775S, Cell Signaling), c-myc (9E10/sc-40, Santa Cruz), myc-tag (71D10/#2278S, Cell Signaling), myc-tag (9B11/#2276S, Cell Signaling), O-GlcNAc (RL2/sc-59624, Santa Cruz), OGT (sc-74546, Santa Cruz), OGA (sc-376429, Santa Cruz), α-spectrin (AA6/MAB1622, Merck), ubiquitin (K48-linkage-specific) (D9D5/#8081, Cell Signaling).

Validation

All antibodies used in this study, were obtained from commercial sources. Information on antibody validation can be obtained from the respective commercial vendors/providers. Additional experimental validation has been performed in-house via immunoblotting experiments using overexpression and/or knock-out/knockdown models or (in case of PTM-specific antibodies) directed pharmacological treatments for the following antibodies: Ataxin-3 (1H9/MAB5360, Merck), CAPN1 (ab39170, Abcam), CAPN2 (1E1F10/66977-1-Ig, Proteintech), CAPN2 (ab39165, Abcam), CAST (#4146S, Cell Signaling), CD99L2 (C-terminal) (HPA061400, Sigma-Aldrich), CD99L2 (N-terminal) (HPA038783, Sigma-Aldrich), FLAG (F7425, Sigma-Aldrich), LC3B (#2775S, Cell Signaling), myc-tag (9E10/sc-40, Santa Cruz), myc-tag (71D10/#2278S, Cell Signaling), myc-tag (9B11/#2276S, Cell Signaling), O-GlcNAc (RL2/sc-59624, Santa Cruz), OGT (sc-74546, Santa Cruz), ubiquitin (K48-linkage-specific) (D9D5/#8081, Cell Signaling).

Eukaryotic cell lines

Policy information about cell lines and Sex and Gender in Research

Cell line source(s)

HEK 293T (293T) cells (ATCC: CRL-11268), SH-SY5Y cells (ATCC: CRL-2266) and primary fibroblasts

Authentication

Authentication of purchased cell lines relied on the provider (ATCC). Additional validation of cell lines was performed by the in-house cytogenetics facility.

Mycoplasma contamination

Used cell lines were tested negative for mycoplasma contamination.

Commonly misidentified lines
(See ICLAC register)

None of the employed cell lines is listed as misidentified by the ICLAC (based on Register version 13, released 26 April 2024).

Clinical data

Policy information about clinical studies
All manuscripts should comply with the ICMJE guidelines for publication of clinical research and a completed CONSORT checklist must be included with all submissions.

Clinical trial registration

NCT04731857

Study protocol

Study protocol for retrospective data analysis can be provided upon request in German.

Data collection

Data were collected in a clinical setting from 2016 to 2024

Outcomes

Firm diagnoses after different genetic testing strategies

Plants

Seed stocks

Report on the source of all seed stocks or other plant material used. If applicable, state the seed stock centre and catalogue number. If plant specimens were collected from the field, describe the collection location, date and sampling procedures.

Novel plant genotypes

Describe the methods by which all novel plant genotypes were produced. This includes those generated by transgenic approaches, gene editing, chemical/radiation-based mutagenesis and hybridization. For transgenic lines, describe the transformation method, the number of independent lines analyzed and the generation upon which experiments were performed. For gene-edited lines, describe the editor used, the endogenous sequence targeted for editing, the targeting guide RNA sequence (if applicable) and how the editor was applied.

Authentication

Describe any authentication procedures for each seed stock used or novel genotype generated. Describe any experiments used to assess the effect of a mutation and, where applicable, how potential secondary effects (e.g. second site T-DNA insertions, mosiacism, off-target gene editing) were examined.
